# Structure and Function of the Su(H)-Hairless Repressor Complex, the Major Antagonist of Notch Signaling in *Drosophila melanogaster*

**DOI:** 10.1371/journal.pbio.1002509

**Published:** 2016-07-12

**Authors:** Zhenyu Yuan, Heiko Praxenthaler, Nassif Tabaja, Rubben Torella, Anette Preiss, Dieter Maier, Rhett A. Kovall

**Affiliations:** 1 Department of Molecular Genetics, Biochemistry and Microbiology, University of Cincinnati College of Medicine, Cincinnati, Ohio, United States of America; 2 Universität Hohenheim, Institut für Genetik, Stuttgart, Germany; 3 Centre for Molecular Informatics, Department of Chemistry, University of Cambridge, Cambridge, United Kingdom; Baylor College of Medicine, UNITED STATES

## Abstract

Notch is a conserved signaling pathway that specifies cell fates in metazoans. Receptor-ligand interactions induce changes in gene expression, which is regulated by the transcription factor CBF1/Su(H)/Lag-1 (CSL). CSL interacts with coregulators to repress and activate transcription from Notch target genes. While the molecular details of the activator complex are relatively well understood, the structure-function of CSL-mediated repressor complexes is poorly defined. In *Drosophila*, the antagonist Hairless directly binds Su(H) (the fly CSL ortholog) to repress transcription from Notch targets. Here, we determine the X-ray structure of the Su(H)-Hairless complex bound to DNA. Hairless binding produces a large conformational change in Su(H) by interacting with residues in the hydrophobic core of Su(H), illustrating the structural plasticity of CSL molecules to interact with different binding partners. Based on the structure, we designed mutants in Hairless and Su(H) that affect binding, but do not affect formation of the activator complex. These mutants were validated in vitro by isothermal titration calorimetry and yeast two- and three-hybrid assays. Moreover, these mutants allowed us to solely characterize the repressor function of Su(H) in vivo.

## Abstract 2

The transcription factor CSL regulates gene expression in response to Notch pathway signaling. The X-ray structure of the complex between the fruit fly version of CSL, Su(H), and its antagonist, Hairless, reveals a novel binding mode and unanticipated structural plasticity.

## Author Summary

Notch signaling is a form of cell-to-cell communication, in which extracellular receptor-ligand interactions ultimately result in changes in gene expression. The Notch pathway is highly conserved from the model organism *Drosophila melanogaster* to humans. When mutations occur within Notch pathway components, this often leads to human disease, such as certain types of cancers and birth defects. Transcription of Notch target genes is regulated by the transcription factor CSL (for CBF1/RBP-J in mammals, Su(H) in *Drosophila*, and Lag-1 in *Caenorhabditis elegans*). CSL functions as both a transcriptional activator and repressor by forming complexes with coactivator and corepressor proteins, respectively. Here we determine the high-resolution X-ray structure of Su(H) (the fly CSL ortholog) in complex with the corepressor Hairless, which is the major antagonist of Notch signaling in Drosophila. The structure unexpectedly reveals that Hairless binding results in a dramatic conformational change in Su(H). In parallel, we designed mutations in Su(H) and Hairless based on our structure and showed that these mutants are defective in complex formation in vitro and display functional deficiencies in in vivo assays. Taken together, our work provides significant molecular insights into how CSL functions as a transcriptional repressor in the Notch pathway.

## Introduction

The Notch pathway is a highly conserved cell-to-cell signaling mechanism that is essential for cell fate decisions during embryogenesis and postnatal tissue homeostasis [1]. In humans, aberrant Notch signaling underlies the pathogenesis of many diseases, including certain types of cancer [2], congenital syndromes [3], and cardiovascular defects [3]. The involvement of Notch in human disease has led to considerable efforts to identify pharmaceuticals that modulate Notch signaling for therapeutic purposes [2].

**Fig 1:**
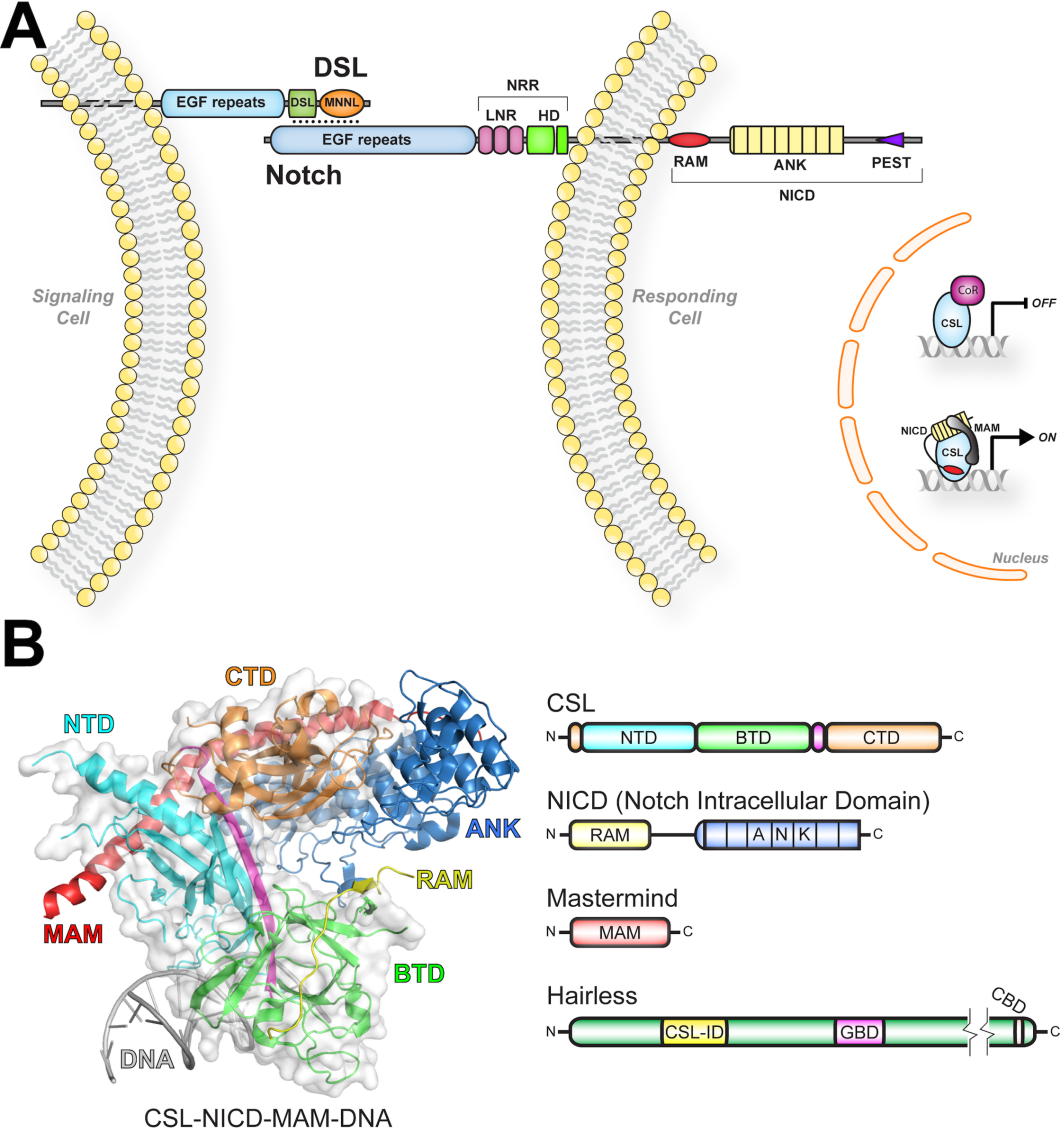
Overview of Notch signaling and structure of the CSL-NICD-MAM activator complex. (A) Figure summarizes canonical Notch signaling. DSL ligands and Notch receptors are multidomain transmembrane containing proteins. Notch-DSL interactions spanning neighboring cells trigger proteolytic cleavage of Notch, generating the Notch intracellular domain (NICD). NICD localizes to the nucleus where it forms a ternary complex with the DNA binding protein CSL and the transcriptional coactivator Mastermind (MAM), to activate transcription from Notch target genes. In the absence of NICD, CSL binds corepressor proteins (CoR) to repress transcription from Notch responsive sites. DSL (Delta, Serrate, Lag-2), MNNL (Module at N-terminus of Notch Ligands), NRR (Negative Regulatory Region), LNR (LIN12-Notch Repeats), HD (heterodimerization domain), RAM (RBP-J associated molecule), and ANK (ankyrin repeats). (B) Structure of the CSL-NICD-MAM ternary complex bound to DNA (PDB ID: 2FO1) [8]. The NTD, BTD, and CTD domains of CSL are colored cyan, green, and orange, respectively. A β-strand that makes hydrogen-bonding interactions with all three domains is colored magenta. The RAM and ANK domains of NICD are colored yellow and blue, respectively. MAM and DNA are colored red and gray, respectively. Domain schematics are colored similarly to the structure. NTD (N-terminal domain), BTD (β-trefoil domain), CTD (C-terminal domain), CSL-ID (CSL interaction domain), GBD (Groucho binding domain), and CBD (C-terminal binding protein binding domain).

The central components consist of the receptor Notch, the ligand DSL (Delta, Serrate, Lag-2), and the nuclear effector CSL (CBF1/RBP-J, Su(H), Lag-1) (Fig 1A) [4]. DSL and CSL are initialisms for the mammalian, fly, and worm orthologous proteins, respectively. Notch and DSL are multidomain transmembrane proteins with a single transmembrane spanning region, and CSL is a DNA binding transcription factor [4]. As shown in Fig 1A, signaling occurs when Notch and DSL molecules on neighboring cells interact, which results in cleavage of Notch and release of the Notch intracellular domain (NICD) from the cell membrane [1]. Subsequently, NICD localizes to the nucleus and directly binds CSL and the coactivator Mastermind (MAM), forming the transcriptionally active CSL-NICD-MAM ternary complex [4]. CSL-NICD-MAM binds at the promoter and enhancer regions of Notch target genes, recruits coactivators, such as p300/CBP and Mediator [5,6], and up-regulates transcription at these sites [7]. Signaling is terminated when NICD is phosphorylated within its PEST domain, resulting in its ubiquitin-mediated degradation [5].

At the structural level, much is known about CSL and the activator complex it forms with NICD and MAM [8–12]. As shown in Fig 1B, the structure of CSL consists of three domains: NTD (N-terminal domain), BTD (β-trefoil domain), and CTD (C-terminal domain) [11]. The NTD and CTD are immunoglobulin (Ig) domains structurally related to the Rel family of transcription factors (e.g., NF-κB and NFAT). CSL binds DNA as a monomer (e.g., CGTGGGAA), in which its NTD and BTD specifically bind bases in the major and minor grooves of DNA, respectively. In addition, CSL-NICD-MAM complexes can also bind as cooperative dimers on DNA at SPS (Su(H) paired site) elements [13,14]. The RAM (RBP-J associated molecule) and ANK (ankyrin repeats) domains of NICD bind the BTD and CTD of CSL, respectively. MAM adopts an extended α-helix that binds the CTD-ANK interface and the NTD of CSL [8,10,12].

CSL can also function as a repressor by interacting with transcriptional corepressor proteins [7], such as Hairless in flies [15], and SMRT/HDAC1 associated repressor protein (SHARP) [16,17] (also known as MSX-2 interacting nuclear target [MINT]) and KyoT2 [18] in mammals (Fig 1). Corepressors are components of large multiprotein complexes that contain histone modification activity, which convert the local chromatin into a repressive environment. While biochemical/cellular studies in mammals demonstrate that CSL interacts with corepressors and functions as a repressor [17,19–21], there is limited genetic data on CSL repressor function. On the other hand, there is extensive biochemical, cellular, and genetic data from the model organism *Drosophila melanogaster* demonstrating that Su(H) (Suppressor of Hairless—the fly ortholog of CSL) functions as a transcriptional repressor at Notch target genes [22]. In this case, Su(H) binds the antagonist Hairless (Fig 1B) [15], which in turn interacts with the corepressors Groucho and CtBP (C-terminal binding protein) to repress transcription [23–25]. Previously, we defined the region of Hairless that interacts with Su(H) and showed that it binds with low nanomolar affinity to Su(H) [26]. Nonetheless, the structural details of Su(H)-Hairless interactions are unknown.

Here, we determine the 2.14 Å X-ray structure of the Su(H)-Hairless repressor complex bound to DNA. As predicted from our previous studies [26], Hairless binds exclusively to the CTD of Su(H), but does so in a strikingly unusual manner. Hairless wedges itself between the two β-sheets that compose the Ig fold of the CTD, significantly distorting the overall fold of this domain. This results in Hairless largely interacting with residues that form the hydrophobic core of the CTD. We designed site-directed mutations to validate our structure and identify the residues critical for Su(H)-Hairless complex formation. Moreover, we were able to design Su(H) mutants that largely affect Hairless binding, but not NICD or MAM, which allowed us to solely characterize its repressor function in cellular assays and in flies. Taken together, our studies provide significant molecular insights into how the antagonist Hairless interacts with the transcription factor Su(H), reveal the remarkable structural plasticity of CSL molecules, and identify a novel binding cleft on the CTD of CSL that could potentially be exploited for modulating Notch signaling.

## Results

### High Resolution Structure of the Su(H)-Hairless Repressor Complex

To determine the Su(H)-Hairless-DNA crystal structure, we purified recombinant Su(H) (98–523) and Hairless (232–269) proteins from bacteria and stoichiometrically formed a complex with a 15-mer duplex DNA, containing a single Su(H) binding site. Su(H) (98–523) corresponds to the structural core of CSL proteins (NTD, BTD, CTD) [11] and Hairless (232–269) comprises the conserved CSL-ID previously shown to be sufficient for Su(H) binding [26]. While crystals were obtained of the Su(H)-H-DNA complex, the crystals diffracted weakly, precluding structure determination. We took two approaches to improve the diffraction properties of our complex crystals: (1) we introduced surface entropy reduction (SER) mutations [27] into our Su(H) construct (R155T and N281G); and (2), we employed a fixed-arm carrier approach [28], in which Hairless (232–269) was purified as a maltose binding protein (MBP) fusion protein (MBP-H). Subsequently, we were able to isolate Su(H)/MBP-H/DNA crystals that diffract to 2.14 Å at a synchrotron source and belong to the space group C2. The Su(H)/MBP-H/DNA complex structure (PDB ID: 5E24) was solved by molecular replacement and refined to a final R factor and free R factor of 17.5% and 19.6%, respectively (Table 1). The contents of the asymmetric unit and representative electron density from the complex structure are shown in S1 Fig. In subsequent figures, which illustrate the details of the Su(H)-Hairless complex, the MBP moiety is not shown for clarity.

**Table 1:** Data collection and refinement statistics.

**Data Collection Statistics**	
Resolution (Å)	31.3–2.14 (2.15–2.14)
Space Group	C2
Wavelength (Å)	0.97872
Unit Cell a, b, c (Å)	177.7, 93.9, 154.4
Unit Cell α, β, γ (°)	90.00, 109.8, 90.00
R_merge_	0.11 (0.65)
I/σI	12.5 (3.1)
Completeness (%)	99.9 (99.8)
Redundancy	7.6 (7.7)
**Refinement Statistics**	
R_work_/R_free_ (%)	0.175 / 0.196
Number of reflections	131,091
Number of atoms: Protein/DNA	13,766
Number of atoms: Ligand/Ion	117 (115 EDO, 2MTT)
Number of atoms: Water	681
B factors: Protein/DNA	53.18
B factors: Ligand/Ion	62.74
B factors: Water	47.81
RMSD Bond Lengths (Å)	0.01
RMSD Bond Angles (°)	1.04
Ramachandran (favored/outliers)	97.44% / 0.30%

Highest resolution shell shown in parentheses.

**Fig 2:**
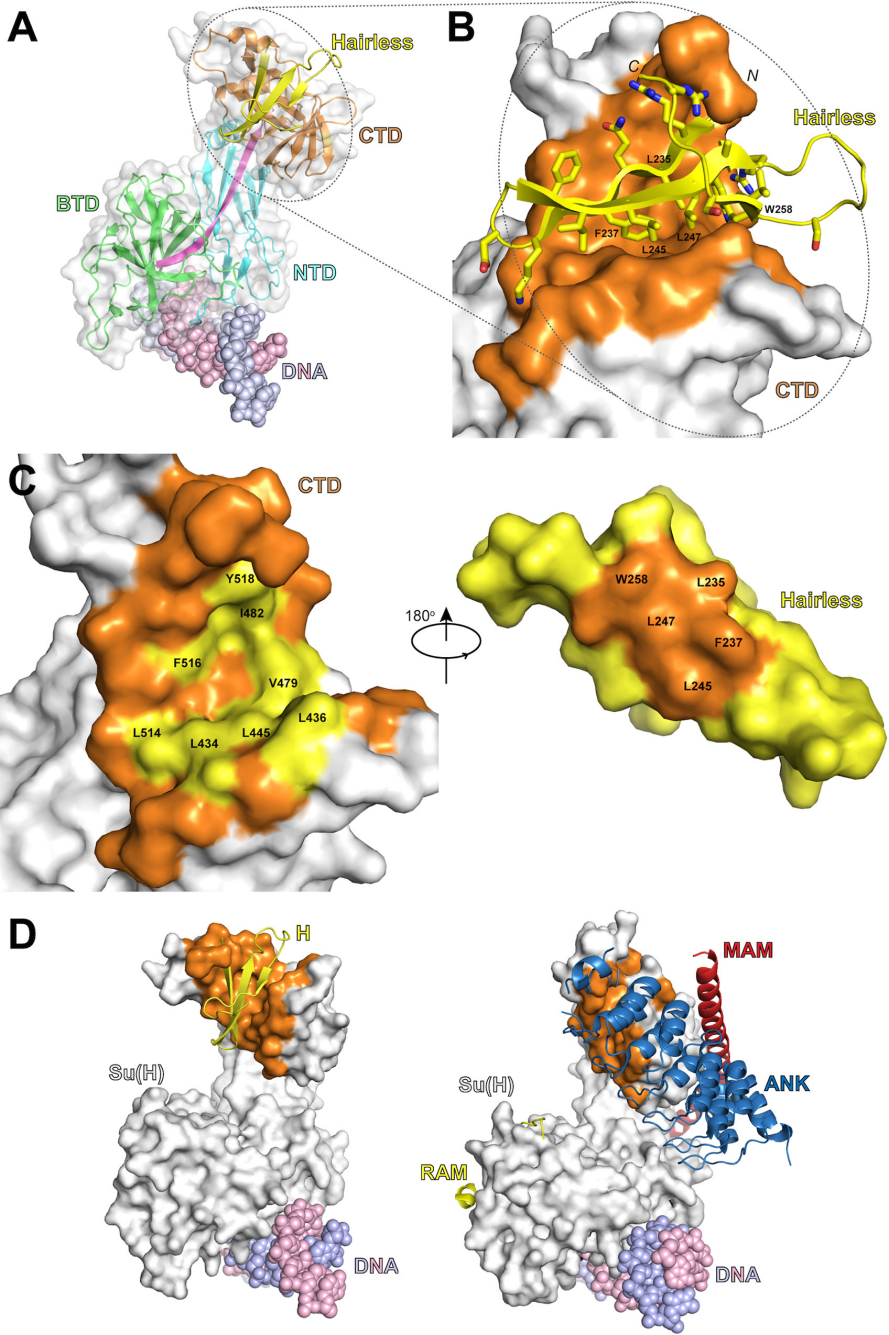
Structure of the Su(H)-Hairless repressor complex bound to DNA. (A) Ribbon diagram of the Su(H)-Hairless structure (PDB ID: 5E24). The NTD, BTD, and CTD are colored similar to Fig 1. Hairless is colored yellow, and the DNA is colored purple and pink. (B) Magnified view of the Su(H)-Hairless interaction. Su(H) is shown as a molecular surface with residues that contact Hairless colored orange. Hairless is shown as cα trace with side chains that directly contact the CTD shown in stick representation. Hairless residues that were mutated in this study are labeled. (C) Open book representation of the Su(H)-Hairless complex. *Left*, Su(H) is shown as a molecular surface with its Hairless binding site colored orange and yellow. Su(H) residues that were mutated here are colored yellow and labeled. *Right*, molecular surface representation of Hairless colored yellow and orange. Residues mutated here are colored orange and labeled. (D) Side-by-side comparison of Su(H)-Hairless repression complex (*left*) and the CSL-NICD-MAM activation complex (*right*, PDB ID: 3V79) [12], which illustrates the partially overlapping binding sites of Hairless, and NICD and MAM on CSL. CSL is represented as a gray surface with the residues that contact Hairless colored orange. Hairless is colored yellow; the RAM and ANK domains of NICD are colored yellow and blue; MAM is colored red; and the DNA is colored purple and pink.

**Fig 3:**
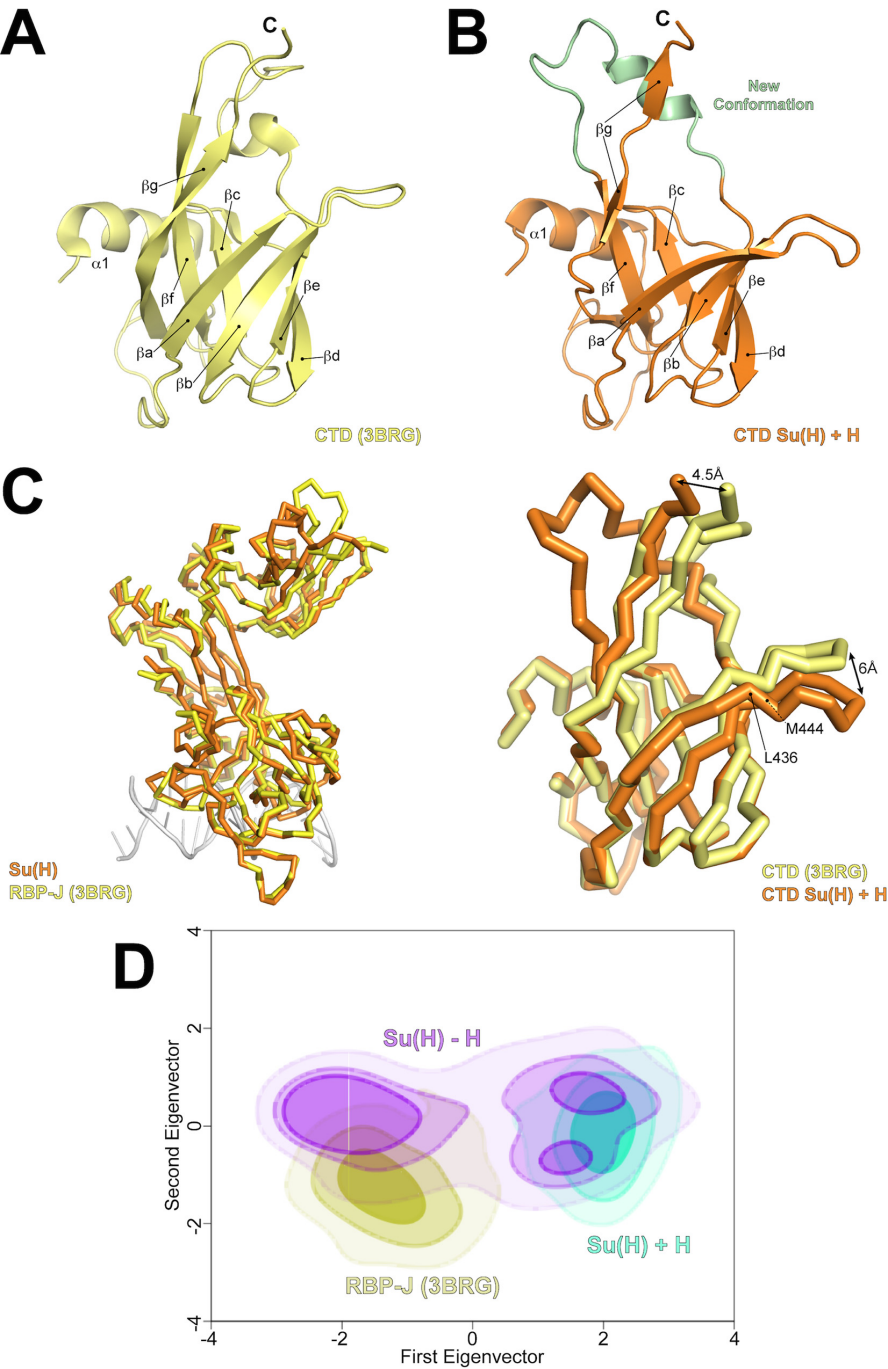
Conformational changes in Su(H) as a result of Hairless binding. (A) Figure shows Ig domain fold of the CTD from the *apo* structure of mouse CSL (RBP-J) (PDB ID: 3BRG) [9], which is composed of seven β -strands (a through g), wherein strands a, b, d, and e form one-half of the β-sandwich, and strands f, g, and c form the other half. (B) Figure shows conformation of the CTD when Hairless is bound. Hairless binds between strands a and g, distorting the overall fold. The largest conformational change occurs in the loop region between strands e and f (colored green). (C) Structural overlay of the cα traces from the *apo* RBP-J structure (3BRG) with the structure of Su(H) from the Su(H)-Hairless complex. *Left*, overall alignment of RBP-J (yellow) with Su(H) (orange). Note the good structural correspondence between the NTD and BTD, but poor structural similarity between the CTD domains. *Right*, zoomed view of the overlay of the CTDs from *apo* RBP-J (yellow) and Su(H) (orange), highlighting the different conformations. The β-hairpin loop between strands a and b (L436-M444) and the C-terminus of the CTD from the Su(H)-Hairless complex undergo a translation of 6 Å and 4.5 Å, respectively, when compared to the structure of *apo* CSL. (D) Principal component analysis of the MD simulations for the Su(H)-Hairless structure (+H, *cyan*), the structure of Su(H) when Hairless is removed (-H, *magenta*), and an *apo* CSL structure *(yellow*). The covariance matrix of the trajectories were constructed, based on the 3-D fluctuations of cα atoms from their average position, and diagonalized, creating eigenvectors and eigenvalues that represent the direction and the amplitude of the motion, respectively. All the trajectories were projected on the first two eigenvectors and eigenvalues of these trajectories.

As shown in Figs 2 and 3, Hairless binds the CTD of Su(H), severely perturbing the overall fold of CTD when compared to an *apo* structure of mouse CSL (also known as RBP-J) [9]. It should be mentioned that there are no *apo* structures of Su(H) solved, necessitating the comparison with the *apo* RBP-J structure. However, given the high degree of sequence similarity between fly and mouse CSL proteins (S2 Fig), we reason that the *apo* RBP-J structure is a good approximation of the *apo* Su(H) structure. For example, within the structural core of CSL, the primary sequence of Su(H) and RBP-J are 82% identical (90% similar); and within the CTD, fly and mouse orthologs are 75% identical (88% similar). Moreover, of the 33 residues in the CTD that are different between Su(H) and RBP-J, 27 of these are surface exposed, likely having minimal effects on folding; and of the remaining 6 residues that are either partially or entirely buried in the CTD, there are only conservative differences between fly and mouse (e.g., Leu→Met, Thr→Val, Ile→Val, and Ser→Thr) (S2 Fig). Nonetheless, in the absence of an *apo* Su(H) structure we cannot rule out minor differences in structure between mouse and fly CSL orthologs.

The Ig-fold of CTD is a seven-stranded β-sandwich, in which four β-strands (a, b, e, d) form half of the sandwich, and the remaining three strands (c, f, g) form the other half (Fig 3A and 3B). Strikingly, Hairless wedges itself between the first and last strands of the β-sandwich (Fig 2A and 2B), making extensive interactions with the hydrophobic core of the CTD and burying ~900 Å^2^ in surface area. For example, Hairless interacts with 10 residues in the CTD that have less than 20 Å^2^ in solvent accessible surface area in a corresponding *apo* structure of CSL (Fig 2C). The site of Hairless binding is remarkably well conserved in mammalian CSL orthologs (S2 Fig). Hairless binding produces relatively modest changes in the first five β-strands of the CTD, with the exception of the first β-hairpin formed by residues L436-M444, which is translated outward by as much as 6 Å (Fig 3C). However, the region between β-strands e and f undergoes the largest structural change upon Hairless binding (Fig 3B), resulting in a completely new conformation of this loop, as well as displacement of the terminal β-strand by as much as 4.5 Å (Fig 3C). We queried the Dali server [29] to identify similar protein complexes. Given the multitude of Ig structures in the database, it was surprising that the search did not uncover any other structurally related complexes. These data suggest that the Su(H)-Hairless complex represent a heretofore novel interaction mode between an Ig domain and its cognate binding partner.

Previously, we showed that the region of Hairless that binds Su(H) is a random coil in solution [26]; however, Hairless assumes an extended β-hairpin conformation when bound to the CTD of Su(H) (Fig 2A and 2B). Hairless residues G232-R249 form a classic β-hairpin, which is followed by an extended loop structure and a third β-strand that makes an anti-parallel interaction with the β-hairpin (Fig 2B). The Hairless β-hairpin is amphipathic, in which its hydrophobic surface, created by residues L235, F237, L245, L247, and W258, buries itself within the core of the CTD (Fig 2B and 2C). In particular, F237 is buried the deepest, anchoring the interaction between Hairless and Su(H).

To analyze the structural changes that occur within CTD when bound to Hairless, we performed molecular dynamics (MD) simulations of the Su(H)-Hairless-DNA complex structure and compared these results with MD simulations of the *apo* RBPJ-DNA structure (PDB ID: 3BRG) (Fig 3D and S3 Fig) [30]. Two simulations were performed, in which Hairless was removed from the model and the resulting Su(H)-DNA structure was allowed to sample different conformations over the time course of the experiment. Both simulations converged to a root mean square deviation (RMSD) value of ~0.35 nm for the cα atoms (S3 Fig), which was similar to the *apo* CSL-DNA structure simulation. Root mean square fluctuation (RMSF) analysis revealed that the largest changes occurred within the CTD of Su(H) (residues 473–498, the region between β-strands e and f) (S3 Fig), which was expected, given that this is the region in Su(H) that incurs the largest structural change upon Hairless binding. This structural rearrangement results in the CTD assuming a more compact conformation that is similar to the *apo* CSL-DNA structure (S3 Fig). Closer inspection revealed that Su(H) residue F516 undergoes a dramatic change in its side chain dihedral angle (~100°) when comparing bound and unbound structures (S3 Fig). Interestingly, the shift in residue F516 of Su(H) is the result of Hairless residue F237 occupying a similar position deep within the core of the CTD. Moreover, when Hairless is removed from the structure, within the first 2–3 ns of the simulation F516 flips its dihedral angle to a conformation similar to the unbound CSL-DNA conformational state and remains in this position for the rest of the simulation (S3 Fig). We also performed principal component analysis of the simulations (Fig 3D), which revealed Su(H) samples two distinct conformational regions (Fig 3D): (1) when bound to Hairless (colored cyan) or (2) in an unbound state (colored yellow). Interestingly, when Hairless is removed from the complex (colored magenta), very early in the simulations Su(H) moves from the bound to unbound conformational region and doesn’t sample the bound conformational region for the remainder of the simulation. Taken together, these data suggest that Hairless binds and stabilizes a strained conformation of Su(H), which is likely a rare high-energy conformer within the ensemble of Su(H) molecules in solution.

Su(H) interacts with the antagonist Hairless and the coactivators NICD and MAM to repress and activate, respectively, transcription from Notch target genes [15,22]. Previous studies suggest that Hairless and NICD/MAM binding to Su(H) are mutually exclusive [26]. Fig 2D shows a side-by-side comparison of the Hairless binding site mapped onto the surface of CSL for the Su(H)-Hairless repression complex and the CSL-NICD-MAM transcriptional activation complex. Clearly, the binding sites for Hairless and NICD partially overlap, and the conformational changes induced in CTD by Hairless binding would sterically block interactions with ANK and MAM. Thus, the Su(H)-Hairless structure supports a model in which Hairless and NICD/MAM binding to Su(H) are mutually exclusive. However, in spite of their partially overlapping binding sites, we speculated that due to their different modes of binding we could design mutations in Su(H) that primarily affect Hairless binding, but largely leave interactions with NICD unaffected. Characterization of these and other mutants by ITC, cellular assays, and in vivo assays in the fly are described below.

### Binding Studies of Su(H) and Hairless Mutants

**Fig 4:**
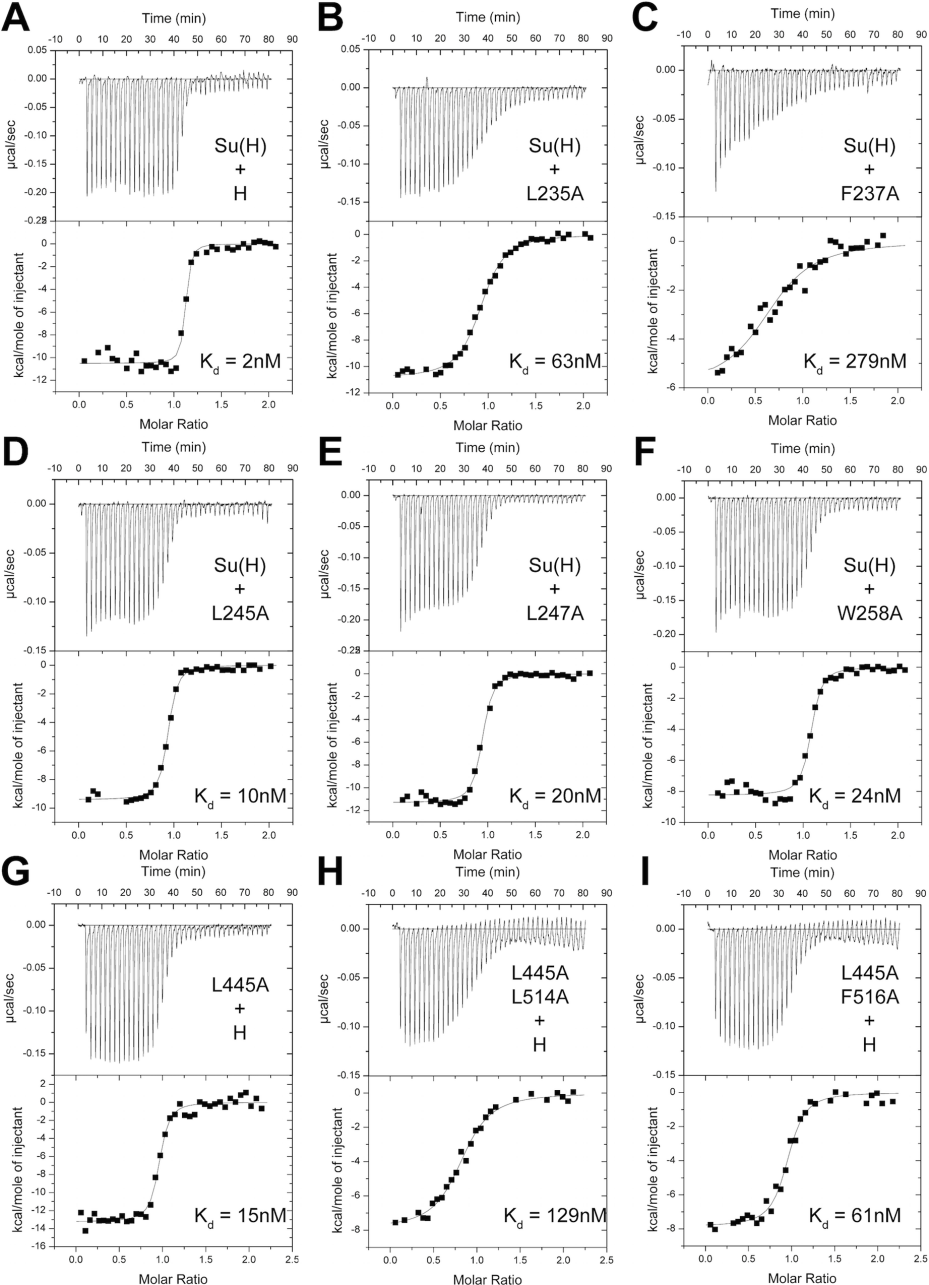
Thermodynamic binding analysis of structure based Hairless and Su(H) mutants. Figure shows representative thermograms (raw heat signal and nonlinear least squares fit to the integrated data) for Su(H) binding to Hairless molecules (wild type and mutants). Each experiment was performed at 25°C, with 40 titrations of 7 μl injections spaced 120 s apart. The dissociation constants (K_d_) shown for each experiment are from Tables 2 and 3. (A) Wild-type Su(H) and Hairless interact with 2 nM affinity. The Hairless point mutants L235A (B) and F237A (C), which have the largest effect on binding, interact with Su(H) with 63 nM and 279 nM affinity, respectively. The Hairless point mutants L245A (D), L247A (E), and W258A (F) have more modest effects on interactions with Su(H), with 10 nM, 20 nM, and 24 nM dissociation constants, respectively. The Su(H) point mutants L445A (G) and L514A have minor effects on binding to Hairless, whereas the Su(H) double mutants L445A/L514A (H) and L445A/F516A (I) have considerable reduced binding to Hairless with 129 nM and 61 nM affinity, respectively.

To analyze the contributions individual residues make to the Su(H)-Hairless complex, we designed mutations in Su(H) and Hairless, based on the structure, and tested the effect these mutations had on binding using ITC. The thermodynamic binding parameters (ΔG°, ΔH°, TΔS°) of these interactions are contained within Tables 2 and 3. As shown Fig 4A (Table 2), Hairless strongly binds Su(H) with 2 nM affinity. Consistent with Hairless being a random coil in solution prior to interacting with Su(H), complex formation is enthalpically driven at 25°C (Table 2). We made alanine substitutions at Hairless residues L235, F237, L245, L247, and W258, which are buried at the interface with Su(H) (Fig 2B and 2C), and determined their affinity for Su(H). H^L235A^ and H^F237A^ affect binding ~30-fold and 140-fold, respectively (Fig 4B and 4C and Table 2), consistent with these residues burying the most amount of surface area at the Su(H)-Hairless interface. H^L245A^, H^L247A^, and H^W258A^, whose side chains are more surface exposed, reduce binding to a lesser extent, 5–12-fold (Fig 4D–4F and Table 2).

**Table 2:** ITC data for the binding of Hairless mutants to Su(H).

*Cell*	*Syringe*	*BSA (Å*^*2*^*)*	*K (M*^*-1*^*)*	*K*_*d*_ *(μM)*	*ΔG° (kcal/mol)*	*ΔH° (kcal/mol)*	*-TΔS° (kcal/mol)*	*ΔΔG° (kcal/mol)*
Su(H)	Hairless (H)	n/a	5.7 ± 1.7 x 10^8^	0.002	-11.9 ± 0.2	-11.9 ± 0.4	-0.05 ± 0.6	n/a
Su(H)	H^L235A^	71	1.7 ± 0.4 x 10^7^	0.063	-9.8 ± 0.2	-9.6 ± 1.1	-0.2 ± 1.3	2.1
Su(H)	H^F237A^	83	4.3 ± 1.9 x 10^6^	0.279	-9.0 ± 0.3	-4.4 ± 1.4	-4.6 ± 1.7	2.9
Su(H)	H^L245A^	56	1.1 ± 0.4 x 10^8^	0.010	-10.9 ± 0.2	-9.3 ± 0.4	-1.6 ± 0.3	1.0
Su(H)	H^L247A^	34	5.1 ± 0.6 x 10^7^	0.020	-10.5 ± 0.1	-11.1 ± 0.4	0.6 ± 0.4	1.4
Su(H)	H^W258A^	78	4.3 ± 0.6 x 10^7^	0.024	-10.4 ± 0.1	-8.5 ± 0.2	-1.9 ± 0.3	1.5

ITC data for the binding of Hairless mutants to Su(H). All experiments were performed at 25°C. Table values are the mean of at least three independent experiments and errors represent the standard deviation of multiple experiments. BSA refers to the amount of surface area buried by the side chain in the native complex structure.

**Table 3:** ITC data for the binding of Su(H) mutants to Hairless and NICD.

*Cell*	*Syringe*	*K (M*^*-1*^*)*	*K*_*d*_*(μM)*	*ΔG° (kcal/mol)*	*ΔH° (kcal/mol)*	*-TΔS° (kcal/mol)*	*ΔΔG° (kcal/mol)*
Su(H)	Hairless	5.7 ± 1.7 x 10^8^	0.002	-11.9 ± 0.2	-11.9 ± 0.4	-0.05 ± 0.6	n/a
Su(H)^L445A^	Hairless	6.7 ± 0.4 x 10^7^	0.015	-10.7 ± 0.1	-12.8 ± 0.4	2.1 ± 0.4	1.2
Su(H)^L514A^	Hairless	1.8 ± 0.5 x 10^8^	0.006	-11.2 ± 0.2	-10.6 ± 0.8	-0.6 ± 1.0	0.7
Su(H)^L445A/L514A^	Hairless	1.0 ± 0.7 x 10^7^	0.129	-9.5 ± 0.4	-7.7 ± 0.7	-1.8 ± 0.6	2.4
Su(H)^L445A/F516A^	Hairless	1.8 ± 0.5 x 10^7^	0.061	-9.9 ± 0.2	-7.9 ± 0.5	-2.0 ± 0.6	2.0
NICD	Su(H)	1.9 ± 0.9 x 10^7^	0.060	-9.9 ± 0.2	-23.8 ± 0.5	13.9 ± 0.5	n/a
NICD	Su(H)^L445A^	1.3 ± 0.2 x 10^7^	0.075	-9.7	-21.6 ± 0.4	11.9	0.2
NICD	Su(H)^L514A^	9.7 ± 0.1 x 10^6^	0.103	-9.6	-18.6 ± 0.3	9.0	0.3
NICD	Su(H)^L445A/L514A^	1.2 ± 0.1 x 10^7^	0.083	-9.7	-16.7 ± 0.2	7.0	0.2
NICD	Su(H)^L445A/F516A^	1.3 ± 0.1 x 10^7^	0.078	-9.7	-16.9 ± 0.2	7.2	0.2

ITC data for the binding of Su(H) mutants to Hairless and NICD. All Su(H)-Hairless binding experiments were performed at 25°C. Table values are the mean of at least three independent experiments and errors represent the standard deviation of multiple experiments. For native Su(H)-NICD binding studies, the table values are from our publication Contreras et al. 2015, and are the mean of at least three independent experiments and errors represent the standard deviation of multiple experiments. For the NICD binding experiments with Su(H) mutants, the errors represent the standard deviation of the nonlinear least squares fit of the data to the titration curves and the table values represent ΔG_obs_, ΔH_obs_, and -TΔS_obs_. n/a = not applicable.

**Fig 5:**
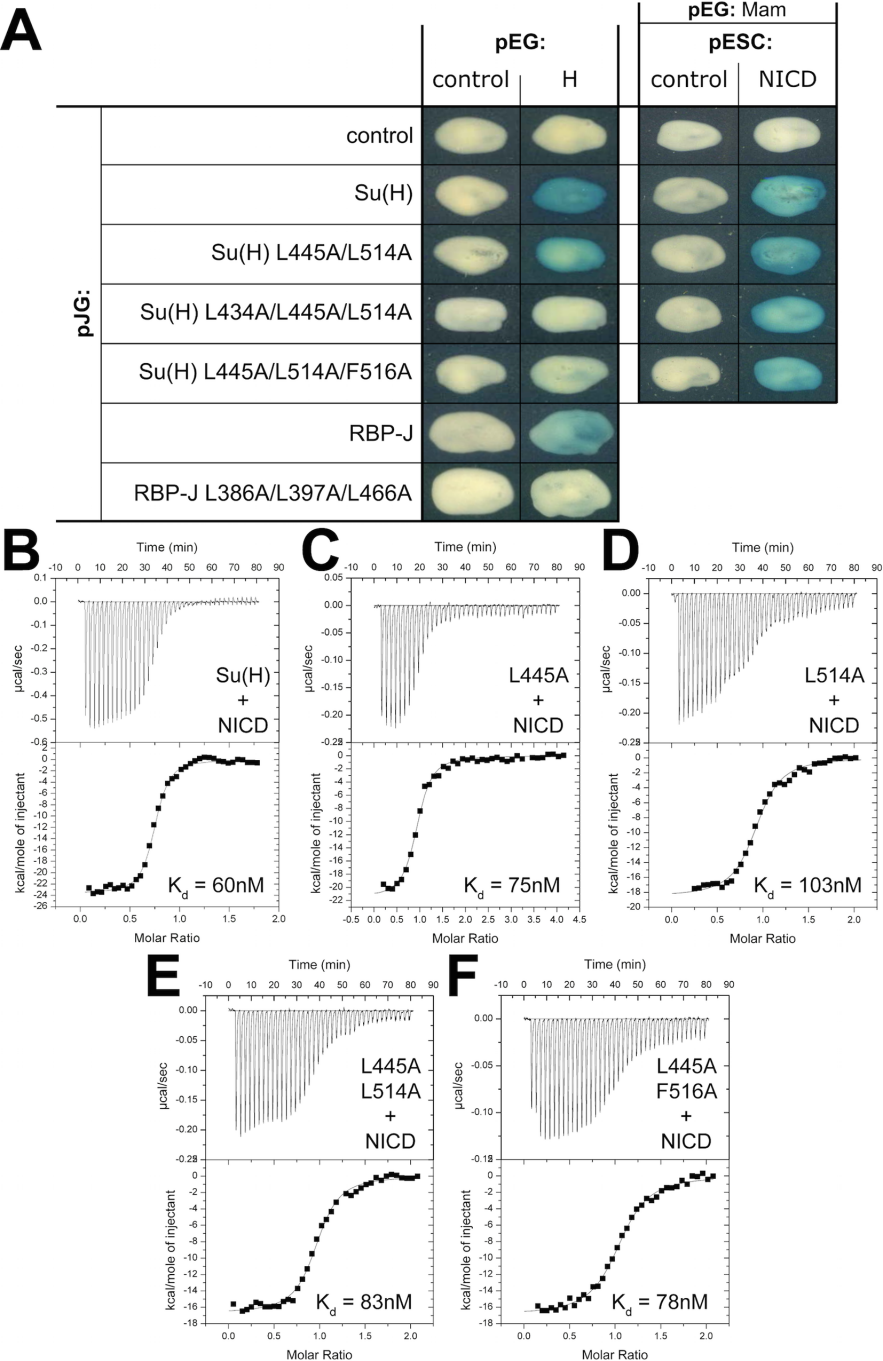
Su(H) double and triple mutants are altered for Hairless binding, but unaffected for binding to NICD. (A) Figure shows yeast two-hybrid results for Hairless (H, 171–357) binding to wild-type and mutant Su(H) and RBP-J constructs. Reduced binding to Hairless is observed for the double mutant Su(H)^L445A/L514A^, whereas the triple mutants (Su(H)^L434A/L445A/L514A^ and Su(H)^L445A/L514A/F516A^) display little to no binding to Hairless. Similar results are observed for RBP-J and RBP-J^L386A/L397A/L466A^. A yeast three-hybrid assay with *Drosophila* MAM (118–194), NICD (1762–2176), and Su(H) shows that the double and triple mutants do not affect formation of the Su(H)-NICD-MAM activator complex. (B–F) Figure shows representative thermograms (raw heat signal and nonlinear least squares fit to the integrated data) for native and mutant Su(H) constructs binding to wild-type NICD (RAMANK, 1762–2142). Each experiment was performed at 25°C, with 40 titrations of 7 μl injections spaced 120 s apart. The dissociation constants (K_d_) shown for each experiment are from Table 3. Su(H) single and double mutants (C–F) have no effect on NICD binding.

Next, we performed alanine-scanning mutagenesis of the residues in Su(H) that contact Hairless (Fig 4G–4I, Table 3 and S1 Table). Some of these residues lie within the hydrophobic core of the CTD, e.g., F460 and I500, and when these were mutated to alanine resulted in insoluble protein, precluding analysis. Alanine substitutions at other sites within the CTD resulted in binding comparable to wild type (S1 Table). However, we were able to identify two mutants, Su(H)^L445A^ and Su(H)^L514A^, which individually only affected binding 8-fold and 3-fold, respectively, but in combination [Su(H)^L445A/L514A^] reduced binding 65-fold (Fig 4G and 4H and Table 3), suggesting that these residues are coupled. We reasoned that side chains near L445 and L514, when substituted for alanine, might also display nonadditive effects on binding. This led us to identify the double mutant Su(H)^L445A/F516A^, which reduced binding to Hairless 31-fold (Fig 4I and Table 3). We also screened numerous combinations of mutants by yeast two-hybrid (Y2H) assays that led to the triple mutants Su(H)^L434A/L445A/L514A^ and Su(H)^L445A/L514A/F516A^, which nearly completely abrogated binding to Hairless (Fig 5A). The Y2H assay also confirmed that the double mutant Su(H)^L445A/L514A^ has reduced binding to Hairless. To support that the Hairless binding site is conserved in mammals, we performed Y2H assays with wild-type and triple-leucine mutant RBP-J molecules. Similar to Su(H), wild-type RBP-J, but not the triple-leucine mutant, binds Hairless (Fig 5A).

We performed several stability and functional assays on the single, double, and triple mutants described above to ensure that these mutant constructs were folded correctly and functionally active for binding to NICD. For the single and double Su(H) mutants, far UV circular dichroism (CD) experiments showed some differences in the spectra between wild-type and mutant proteins; however, analysis of the CD data with Contin-LL [31] revealed similar amounts of secondary structure between native and mutant Su(H) proteins (S4 Fig). Similarly, thermal shift assays showed some destabilization of the mutants compared to wild type, but not substantial misfolding, and EMSA confirmed that the mutants bind DNA comparable to wild-type Su(H) (S4 Fig). As NICD also binds the CTD of Su(H) [26,32], we used ITC to test whether the mutants affect NICD binding. As shown in Fig 5B–5F (Table 3), NICD binds Su(H) with 60 nM affinity, and importantly, the single and double mutants of Su(H) have little to no effect on NICD binding, suggesting that the mutations do not significantly affect the fold or function of the CTD. Unfortunately, we were unable to purify enough protein of the Su(H) triple mutants to analyze these constructs in vitro. However, we do demonstrate that the triple mutants can interact with NICD and MAM in a yeast three-hybrid assay (Fig 5A), again suggesting generally correct folding and function. Taken together, despite their partially overlapping binding sites on Su(H), we have identified mutations in Su(H) that affect complexes with Hairless, but leave interactions with NICD and MAM largely intact; albeit, with the caveat that the mutations do effect the stability of Su(H) to some degree. Henceforth, we refer to these mutants as Su(H)^LL/AA^, Su(H)^LLL/AAA^, and Su(H)^LLF/AAA^, respectively.

### In Vivo Characterization of Su(H) Mutants

**Fig 6:**
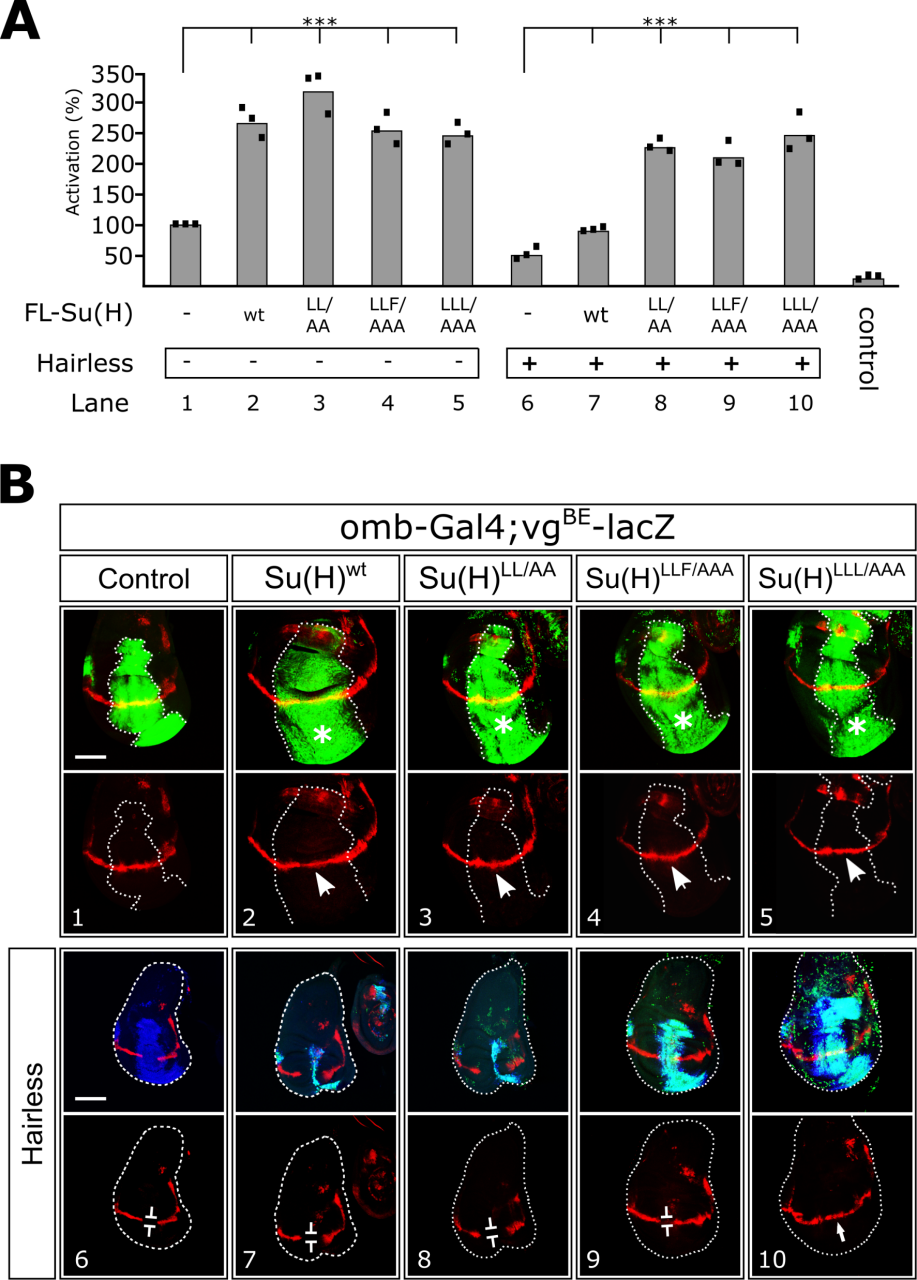
Activity of wild-type and mutant Su(H) isoforms in cellular Notch reporter assays and in vivo in *Drosophila*. (A) S2 cells were cotransfected with the NRE luciferase reporter, NICD, and the indicated constructs of Su(H), with or without Hairless. Reporter activity was taken as 100% for cells transfected with NICD (lane 1). Three independent experiments were performed as indicated. Statistical significance was assessed using Dunnett’s test with *** *p* ≤ 0.001. The underlying data can be found in S1 Data. (B) Figure shows in vivo assays for formation of the activator and repressor complex in the wing imaginal discs of stage-matched larvae. Expression from the *vg*^*BE*^*–lacZ* reporter is colored red and was determined in the presence of ectopically expressed UAS-Su(H) constructs (green) as indicated, either singly (*panels 1–5*) or in combination with ectopically expressed Hairless (UAS-H, *panels 6–10*). All constructs were overexpressed in the central domain of the wing anlagen using the omb-Gal4 driver (encircled with a dashed line, Su(H) is colored green). UAS-GFP served as control (*panel 1*). Hairless is colored blue and in overlays with Su(H) appears turquoise (*third row*). Overexpression of Su(H) isoforms causes a subtle induction of the *vg*^*BE*^*–lacZ* reporter, denoted by arrows, and an overproliferation of the affected tissue (asterisks, *panels 2–5*). Concomitant overexpression of Su(H) isoforms and Hairless (*panels 7–10*) results in repression of the *vg*^*BE*^*–lacZ* reporter, denoted by bars, and tissue loss, as observed by loss of green and blue staining (*panels 6–8*). Discs were framed to visualize the morphology in lower panels. Size bars = 100 μm.

To test our Su(H) mutants within cells, we transfected *Drosophila* Schneider S2 cells with the NRE (Notch Response Element) reporter, which contains Su(H) binding sites coupled to the luciferase gene [33]. S2 cells express Su(H) and Hairless, but require the cotransfection of NICD to activate the reporter (Fig 6A) [23,33]. Providing additional Su(H) in the cells raises the activity about 3-fold, as observed previously [23,26,34]. Cotransfection of the Su(H) mutant constructs (Su(H)^LL/AA^, Su(H)^LLF/AAA^, Su(H)^LLL/AAA^) result in a very similar increase in reporter activity, in accordance with activator complex (Su(H)-NICD-MAM) assembly on DNA (Fig 6A). To address whether the repressor activity of Su(H) is compromised by the mutations, S2 cells were cotransfected with Hairless. Cotransfection of NICD and Hairless results in a 2-fold loss in reporter activity, which is restored by transfection of Su(H) (Fig 6A) [23,26,34]. Apparently, the competition of NICD and Hairless for Su(H) determines the overall reporter activity level. However, cotransfection of the Su(H) mutants, which are deficient for Hairless binding, result in activation of the reporter similar to cotransfection of Su(H) (native or mutant) and NICD (Fig 6A). We attribute the slight differences in reporter activity to repression mediated by endogenous Su(H) with the addition of exogenous Hairless and/or the mutations having a minor effect on NICD binding and/or residual binding of the mutants to Hairless. Nonetheless, these data suggest that in S2 cells our Su(H) mutants are competent to form an activator complex with NICD and MAM, but are defective for interacting with Hairless.

To address the in vivo activity of our Su(H) mutants, we established transgenic fly lines that allow for tissue-specific overexpression [26]. Insertion at the identical site (96E) avoided unwanted position effects [26,35]. In addition, combined overexpression of Hairless and the Su(H) constructs was made possible by recombination [26]. The capacity of our Su(H) mutants to activate transcription was analyzed with the *vg*^*BE*^*-lacZ* reporter gene, a readout for the Notch target gene *vestigial* [36]. Ectopic expression of the constructs (wild type and mutants) was induced in the central domain of the wing anlagen. In response to Notch activation, the *vg*^*BE*^*-lacZ* reporter is expressed along the presumptive wing margin [36] (*red stripe*, Fig 6B panel 1), which can be easily scored for differences in Notch signaling. Similar to ectopic expression of wild-type Su(H), the three mutant Su(H) constructs (Su(H)^LL/AA^, Su(H)^LLF/AAA^, Su(H)^LLL/AAA^) result in a weak expansion of *vg*^*BE*^*-lacZ* expression (Fig 6B panels 2–5). In addition, activation of Notch, due to ectopic expression of Su(H), causes an overgrowth of the wing disc [23,26,34]. Similar overgrowth is observed for the three Su(H) mutants when compared to wild-type Su(H) (asterisks in Fig 6B panels 2–5), suggesting that the mutations have little to no effect on assembly of the activator complex and activation of a Notch target gene in vivo.

To address whether the Su(H) mutants affect repressor complex formation in vivo, they were coexpressed with Hairless and *vg*^*BE*^*-lacZ* reporter expression was monitored (Fig 6B). As a control, we ectopically expressed Hairless alone and in combination with wild-type Su(H), obtaining the expected results from a down-regulation of Notch signaling. Ectopic Hairless expression strongly repressed the *vg*^*BE*^*-lacZ* reporter, which was accompanied by a loss of tissue in the overexpression domain (Fig 6B panel 6). Combined overexpression of Su(H) and Hairless resulted in widespread silencing of the *vg*^*BE*^*-lacZ* reporter and a nearly complete loss of tissue within the overexpression domain (Fig 6B panel 7), consistent with a hyper-repression of Notch signaling in this tissue [26,34]. This phenotype has been interpreted as a result of ectopic repressor complex formation, reflecting Su(H) and Hairless protein binding in a highly sensitized manner [24,37].

In the case of a complete loss of binding between the Su(H) mutants and Hairless, i.e., independent activity of the two components, the combined overexpression should result in an additive phenotype: (1) activation of Notch signaling by the Su(H) mutant defective in Hairless binding and (2) repression by Hairless. The two should level out each other, and hence, wing discs resembling wild type would be expected. Any residual binding between Su(H) mutants and Hairless, however, would be uncovered by the strong super-repression effect resulting from their combined overexpression. As shown in Fig 6B (panel 10), ectopic expression of the triple mutant Su(H)^LLL/AAA^ with Hairless resulted in normal expression of the *vg*^*BE*^*-lacZ* reporter and the wing disc was similar to the control, suggesting an almost complete loss of Hairless binding by the mutant. Ectopic expression of Su(H)^LLF/AAA^ with Hairless was less effective (Fig 6B panel 9), perhaps reflecting residual binding to Hairless and/or potentially subtle defects in activator complex formation, which our other assays were not sensitive enough to detect. The appreciable binding of the double mutant Su(H)^LL/AA^ to Hairless observed in vitro was uncovered in our in vivo experiment with a repression of Notch activity similar to wild type Su(H) (Fig 6B panel 8).

Finally, these results were confirmed by a phenotypic analysis of adult flies, in which the Su(H) mutant constructs and Hairless were coexpressed during eye development. As shown in S5 Fig, and consistent with our previous results, ectopic induction of all three Su(H) mutants in the eye imaginal discs effects eye morphology indistinguishable from wild-type Su(H), but the triple mutants Su(H)^LLL/AAA^ and Su(H)^LLF/AAA^ retain little to no binding of Hairless in vivo. However, in this in vivo context Su(H)^LL/AA^ retained considerably less Hairless-mediated repressor activity, reflected by a milder phenotype compared to the Su(H)/H co-overexpression.

## Discussion

In the Notch pathway, extracellular interactions are transduced into changes in gene expression via the transcription factor CSL [1]. To activate transcription, CSL forms a ternary complex with the intracellular domain of the Notch receptor (NICD) and the coactivator Mastermind (MAM), which binds at the promoter and enhancer regions of Notch target genes. A number of crystal structures that correspond to the CSL-NICD-MAM complex have been determined, and accompanying functional studies have scrutinized its role as an activator [4]. To repress transcription, CSL binds corepressor proteins, such as Hairless from *Drosophila*, and SHARP or KyoT2 from mammals, which recruit other factors involved in transcriptional repression, such as Groucho, CtBP, NCoR, and Polycomb-group proteins, thus localizing the transcriptional repression machinery to Notch target genes [7]. While CSL is absolutely required to activate transcription at all target genes, its role as a repressor in different organisms is not as clear-cut. In mammals, RBP-J (CSL ortholog in mammals) has been shown to directly bind SHARP [16,17,20] and KyoT2 [18,19], and cellular studies have shown that corepressors and their associated complexes are recruited to Notch target genes [21,38]; for certain cases, loss of RBP-J or its associated corepressor results in the de-repression of some, but not all target genes [21,38,39]. These in vitro data, however, have not yet been fully replicated in vivo, as there a only a few examples in mouse knockout studies that suggest RBP-J functions as a transcriptional repressor [40–42].

On the contrary, in *Drosophila* there is substantial evidence both in vitro and in vivo that Su(H) (CSL ortholog in flies) binds the antagonist Hairless and functions as a transcriptional repressor at Notch target genes [15,22]. To provide a detailed structural basis for this interaction and gain additional insights into its function, here we determined the high-resolution structure of the Su(H)-Hairless repressor complex bound to DNA (Fig 2), and presented convincing in vitro and in vivo mechanistic studies that complement and support our structure (Figs 4–6). The most striking feature of the Su(H)-Hairless complex is the substantial conformational change that occurs in the CTD of Su(H) upon Hairless binding (Fig 3). This results in the two β-sheets that compose the Ig-fold of the CTD to be splayed apart, exposing residues in the hydrophobic core of the CTD that form the basis for the Su(H)-Hairless interaction. To our knowledge, this conformational change and binding mode observed in our Su(H)-Hairless complex structure is without precedence in other Ig domain-containing structures. On the other hand, this illustrates the remarkable structural plasticity of this Ig domain in CSL. It will be interesting to see whether this occurs in other Ig-fold proteins, i.e., whether this binding mode is a basic principle of Ig domains or is particular to CSL proteins.

The structural change in Su(H) upon Hairless binding represents the largest and most striking conformational change observed in all CSL-mediated complex structures determined to date. This raises the question as to what role the structural change plays in Su(H) function. One possibility is that the conformation Su(H) assumes when bound to Hairless serves to recruit specific binding partners or target the Su(H)-Hairless complex to specific sites in the genome. While the identities of these potentially new binding partners are unknown, it would be interesting in future studies to use screening approaches in order to identify factors that only bind the Su(H)-Hairless complex. A similar approach was used previously to identify Mastermind in *Caenorhabditis elegans* [43], a factor that only binds CSL when NICD is present. Moreover, our Su(H) mutants that are defective in Hairless binding may be useful in genome-wide studies to identify DNA sites bound by the Su(H)-Hairless complex.

Our MD simulations of the Su(H)-Hairless complex structure demonstrate that the conformation Su(H) must assume to bind Hairless is energetically unfavorable, and therefore, likely to be a rare conformer in the ensemble of Su(H) molecules in solution. The fact that the region of Hairless that interacts with Su(H) is unstructured prior to complex formation [26] suggests that the kinetics of Su(H)-Hairless association (k_on_) is a relatively slow process. If this is indeed the case, then this would require the off rate (k_off_) to also be relatively slow in order to achieve the 2 nM affinity (K_d_ = k_off_/k_on_) determined for Su(H)-Hairless complexes, which is the strongest CSL binding partner measured to date. While the functional consequences of these slow on/off rates for Su(H)-Hairless complexes are not immediately evident, intriguingly these maybe important for its role as a transcriptional repressor, its subcellular localization, and/or replacement of activation complexes at Notch target genes. In the latter case, given that dimeric Su(H)-NICD-MAM complexes are stabilized at SPS sites by cooperative interactions between the ANK domain of NICD [13,14], it is conceivable that the displacement dynamics between monomeric versus dimeric Notch activation complexes and Su(H)-Hairless repressor complexes at Notch target genes are different. However, to our knowledge, there are no data available to support this hypothesis, but it would be interesting to investigate this in the future.

Regarding the subcellular localization of Su(H), Hairless shuttles Su(H) into the nucleus [44,45], similar to NICD [46,47]. Moreover, there are two examples that demonstrate the amount of nuclear Su(H) is dependent on Hairless: (1) when Hairless is overexpressed, then Su(H) accumulates in the nucleus [44,45]; and (2) when cell clones are lacking Hairless, then Su(H) protein displays a conspicuously lower abundance [45]. Interestingly, an inter-dependence of Notch and Su(H) protein levels has been reported in the *Drosophila* embryo [48]. Perhaps, Hairless and/or NICD binding to Su(H), and the nuclear import of these complexes may protect Su(H) from degradation in the cytosol. In mammals, it has been shown that RBP-J requires interactions with either corepressors or NICD for nuclear localization [49], and RBP-J is subjected to degradation in response to phosphorylation by p38 MAP kinase [50]. Interestingly, these data raise the possibility that a similar mechanism in flies and mammals exists to regulate CSL subcellular localization and turnover.

While several corepressors that interact with RBP-J have been identified in mammals [4,7], there are no Hairless orthologs found outside of insects [15]. Moreover, the mammalian corepressors all interact with the BTD of RBP-J similar to the RAM domain of NICD, which provides a potential mechanism whereby corepressors and NICD could compete for binding to RBP-J. For example, both the corepressor KyoT2 and RAM interact with the BTD of CSL in very similar conformations [19], whereas Hairless exclusively binds the CTD to a newly identified binding cleft on Su(H). Moreover, there are no known corepressors in *Drosophila* that interact with the BTD of Su(H). Interestingly, the corepressor SHARP, which has been suggested to be a Hairless analogue in mammals, interacts with both the BTD and CTD of RBP-J [17,20]. Given the high degree of sequence similarity between fly and mammalian CSL proteins (S2 Fig), perhaps SHARP binds the CTD of RBP-J in a manner similar to Hairless. Nonetheless, future studies that focus on the structure and function of the RBPJ-SHARP corepressor complex will be important for elucidating the enigmatic repressor role RBP-J plays in mammals.

Finally, there have been extensive efforts in the pharmaceutical industry and academic labs to identify reagents that modulate Notch signaling for therapeutic endpoints [2]. For example, γ-secretase inhibitors and monoclonal antibodies that target the extracellular domains of Notch receptors and ligands have been developed to treat certain types of cancer. However, in comparison, there has been very little progress in identifying and developing small molecules that directly target RBP-J and the transcription complexes it forms with coregulators. Our Su(H)-Hairless structure provides a new binding cleft that could be targeted. Consistent with this notion, previous studies from our groups demonstrated that Hairless binds RBP-J and when only the CSL-ID of Hairless is expressed in mammalian cells, it acts as a potent antagonist of Notch signaling in transcriptional reporter assays [26].

## Materials and Methods

### Protein Overexpression and Purification

The codons that correspond to Su(H) residues 98–523, which represents the structural core of CSL (NTD, BTD, CTD), were cloned into pGEX-6P1. Site directed mutagenesis was used to introduce surface entropy reduction (SER) mutations (R155T and N281G) into pGEX-6P-1-Su(H) for purification and crystallization purposes. Wild-type and R155T/N281G Su(H) proteins were demonstrated to have nearly identical binding to Hairless [26]. Similarly, site directed mutagenesis was used to make the Su(H) mutant constructs described herein. As described previously [26], GST-Su(H) was overexpressed in BL21 Tuner cells (Novagen) and purified to homogeneity using a combination of affinity (glutathione resin), ion exchange (SP), and size exclusion chromatography. The purified protein was concentrated, flash frozen in liquid nitrogen, and stored at -80°C in a buffer containing 20 mM MES pH 6.0, 0.5 M NaCl, 1% ethylene glycol, and 0.1 mM TCEP.

The codons that correspond to Hairless (H) residues 232–269, which comprise the conserved CSL-ID previously shown to be sufficient for Su(H) binding [26], were cloned into pMAL-E, which produces an MBP-H fusion protein. The MBP moiety also contains SER mutations to facilitate crystallization [28]. MBP-H was overexpressed in BL21 Tuner cells and purified to homogeneity using affinity (amylose resin) and size exclusion chromatography. The purified protein was concentrated, flash frozen in liquid nitrogen, and stored at -80°C in a buffer containing 20 mM Tris pH 7.4, 0.5 M NaCl, 1 mM EDTA, 1% ethylene glycol, and 5 mM maltose. The codons that correspond to Hairless residues 232–358 were cloned into pSMT3, producing a SMT3-H fusion protein with an N-terminal His tag, which was used for ITC binding studies with Su(H), as described previously [26]. Site-directed mutagenesis was used to introduce Hairless mutants into the pSMT3-H construct. The SMT3-H fusion protein was overexpressed in BL21(DE3) Tuner cells and purified using affinity (Ni-NTA resin) and ion exchange (SP) chromatography. Wild-type and mutant SMT3-H proteins were dialyzed into 50 mM sodium phosphate pH 6.5 and 150 mM NaCl for ITC binding studies.

### Crystallization, Data Collection, and Structure Determination

In order to crystallize and determine the X-ray structure of the Su(H)-Hairless-DNA complex, recombinant Su(H) (98–523) and MBP-Hairless (232–269) proteins were purified to homogeneity from bacteria and stoichiometrically bound to an oligomeric 15-mer duplex DNA, containing a single Su(H) binding site (TTACTGTGGGAAAGA, AATCTTTCCCACAGT). Crystals were grown out of a mother liquor containing 0.1 M Tris pH 8.0, 19% PEG3350, 0.2M (NH_4_)_2_SO_4_, were cryoprotected with 20% ethylene glycol, and were flash frozen in liquid nitrogen for data collection. X-ray diffraction data were collected on frozen crystals (200 K) at the Advanced Photon Source on the LS-CAT beamline 21-ID-F (λ = 0.97872 Å) and processed with XDS [51]. Su(H)/MBP-H/DNA complex crystals belong to space group C2 with unit cell dimensions (a = 177.7, b = 93.9, c = 154.4, β = 109.8°) (Table 1). The Su(H)/MBP-H/DNA structure was solved using molecular replacement (Phaser) [52] with the search models 3BRG [9] and 3H4Z [28], which contain RBP-J bound to DNA and MBP, respectively. The asymmetric unit of the crystals contains two Su(H), two MBP-H, and one DNA duplex (S1 Fig). The Hairless structure was built manually with COOT [53], and the structure was refined with Phenix [54] and Buster [55] to a final R factor and free R factor of 17.0% and 19.4%, respectively (Table 1). The model was evaluated with Molprobity [56] and the Ramachandran statistics for the final structure are 97.2% of the residues in the favored region with 0.24% as outliers.

The structure indicates that Hairless residues G232-R263 form the core structural motif that interacts with Su(H), whereas residues K264-P269 neither contact Su(H) nor the β-hairpin motif of Hairless, and their extended structure is stabilized by interactions with the crystal lattice. Representative electron density of the CTD-Hairless interaction is shown in S1 Fig. The asymmetric unit (AU) of the crystals contains two Su(H)/MBP-H complexes, but surprisingly, only one of the complexes is bound to DNA (S1 Fig). While the explanation for the crystallization of these asymmetric complexes is unclear, the overall conformation of Su(H)-Hairless in the two complexes is similar (RMSD 0.92 for 442 cαatoms). The primary difference lies in the temperature factors (B factors) between the two complexes in the AU, in which the regions in the NTD and BTD that bind DNA have much higher B factors for the complex that is missing DNA (S1 Fig). All structure figures were created with PyMOL [57].

### Isothermal Titration Calorimetry

ITC experiments were carried out using a MicroCal VP-ITC microcalorimeter. All experiments were performed at 25°C in a buffer composed of 50 mM sodium phosphate pH 6.5 and 150 mM NaCl. Su(H) and SMT3-H (Hairless) proteins were degassed and buffer-matched using dialysis and size exclusion chromatography. A typical experiment contained 5 μM Su(H) in the cell and 50 μM SMT3-H in the syringe. The data were analyzed using ORIGIN software and fit to a one-site binding model.

### Molecular Dynamics Simulations Analysis

Molecular dynamics simulations of an *apo* CSL structure (PDB ID: 3BRG) and the Su(H)-Hairless complex structure were performed with the AMBER11 package, using the AMBER FF99SB force field [58]. A dodecahedral box of water molecules, treated as in the TIP3P model [59], was built around the complex and a physiological concentration of 0.15 M NaCl was used. For each experiment, the following protocol was used: (1) *in vacuo* minimization (1,000 steps); (2) minimization, keeping the complexes fixed, allowing water molecules and ions to equilibrate (1,000 steps of steepest descent plus 1,000 steps of conjugate gradient); (3) minimization of the entire system, without restrictions (1,000 steps of steepest descent plus 1,000 steps of conjugate gradient); (4) NVT equilibration, 1 ns; and (5) 2 x 100 ns production phase. All calculations were performed with the CUDA-enabled version of PMEMD [60], using TESLA GPUs at the High Performance Computing (HPC) cluster of the University of Cambridge. Analysis of the trajectories was performed with the AMBERTOOLS 1.5, GROMACS [61] and RStudio packages. Cavity analysis of the most representative structures of the principal clusters of the Su(H) trajectory was performed using trj_cavity [62].

### Yeast Two- and Three-Hybrid Assay

The yeast two- and three-hybrid experiments were performed in triplicate as described previously [26,34]. pEG-MAM corresponds to *D*. *melanogaster* Mastermind, comprising codons 118 to 194. pESC-NICD corresponds to the RAM and ANK domains of *Drosophila* Notch, comprising codons 1762 to 2176. pEG-Hairless corresponds to *Drosophila* Hairless, comprising codons 171 to 357. Amino acid substitutions in full-length Su(H) and RBP-J were introduced using QuickChange II XL Site directed Mutagenesis Kit (Agilent) and ultimately cloned into pJG. The three promising mutants (L445A/L514A, L445A/L514A/F516A, L434A/L445A/L514A) were shuttled into pUAST-attB and pMT vectors. All constructs were sequence verified.

### Cellular Reporter Assays

Reporter assays were performed in triplicate as described in Maier et al. [26]. Schneider S2 cells (obtained from the DGRC) were transiently transfected with 1 μg of the NRE-luciferase reporter [33] and 0.2 μg of control Renilla plasmid (Promega) to normalize transfection. pMT-NICD was cotransfected with 0.5 μg of the relevant pMT-Su(H) construct and/or 0.5 μg pMT-Hairless. The total amount of transfected DNA was kept constant at 3 μg by using the pMT-A vector (Promega). Constructs were induced 6 h after transfection by adding 0.5 mM CuSO_4_; 18 h later, cells were harvested and luciferase activity was measured in duplicate with a luminometer (Lumat LB9507), using the dual-luciferase reporter assay system according to the manufacturer’s protocol (Promega). The effects of the addition of respective Su(H) variants relative to the controls were assessed statistically using ANOVA and Dunnett’s test (***p ≤ 0.001).

### In Vivo Analysis of Hairless and Su(H) Transgenes

Integration of the pUAST-attB Su(H) constructs at chromosomal position 96E was done with the help of the PhiC31 integration system, as described previously [26,35]. Several lines were obtained that behaved similarly. Tissue specific overexpression was induced with the driver lines omb-Gal4 and gmr-Gal4 (FlyBase, http://flybase.org). Recombination of the UAS-Su(H) variants with UAS-Hairless HFL at 68E [26] was done with standard genetic protocols and verified by PCR. Crosses were set up multiple times and representative images of stage-matched larvae are shown. Expression of the *vg*^*BE*^*-lacZ* reporter [36] was monitored by antibody staining against β-galactosidase (clone 40-1a; developed by J. R. Sanes; obtained from Development Studies Hybridoma Bank, University of Iowa, IA); expression of the constructs was controlled by appropriate antibody staining using anti-H and anti-Su(H) [44]. Secondary antibodies coupled to FITC, Cy3, and Cy5 were purchased from Jackson ImmunoResearch Laboratory (West Grove, PA). Pictures were taken with a Zeiss Axiophot linked to a confocal microscope (Bio-Rad MRC 1024) using LaserSharp Version 2.0 software. Fly heads were captured with an ES120 camera (Optronics) linked to a Wild stereo microscope and Pixera Viewfinder Version 2.0 software. Phenotypes were consistent over multiple trials; n = 20 heads of each genotype were evaluated.

### Circular Dichroism

CD measurements were taken in triplicate using an Aviv Circular Dichroism Spectrometer model 215 at 25°C in a 0.02 cm cuvette. Wavelength scans were performed between 190 and 240 nm using 1.0 nm increments. Su(H) proteins were in a buffer containing 10 mM Tris-phosphoric acid pH 7.4 and 50 mM NaF. Protein concentrations were between 2–4 mg/ml. CD data were analyzed using Contin-LL (Provencher and Glockner Method) [31] with the SMP180 reference set.

### Thermal Shift Assays

An Applied Biosystems StepOne Real Time PCR system was used to collect the thermal shift data and the data were processed with their proprietary Protein Thermal Shift Software v1.2. Su(H) proteins were used at a concentration of 7 μM in a buffer containing 25 mM MES pH 6.0, 0.5 M NaCl, 1 mM EDTA, and 1 mM TCEP.

### EMSA

EMSAs were performed as described previously [9,26]. Wild-type or mutant Su(H) constructs were bound to an oligomeric 19-mer duplex that contains a single CSL-binding site and separated on a 7% polyacrylamide gel containing 0.5x Tris-borate buffer, pH 7, for several hours at 4°C. Complexes were visualized on the gel using SYBR-GOLD stain (Invitrogen).

## Supporting Information

S1 Data Excel file containing in separate sheets the numerical data for Figs 6A and S4. (XLSX)

S1 Fig Additional details of the Su(H)-Hairless-DNA X-ray structure determination. (A) Figure shows orthogonal views of the molecules contained within the asymmetric unit of the crystal. The asymmetric unit contains two Su(H) molecules (colored yellow and magenta), two MBP-Hairless fusion molecules (colored cyan and green), and one DNA duplex (colored gray). MBP/H-A, MBP/H-B, Su(H)-C, and, Su(H)-D refer to chains A, B, C, and D, respectively, contained within the PDB file. (B) Figure shows a temperature factor (B-factor) comparison of the two Su(H) molecules contained within the asymmetric unit of the crystals. The color gradient from blue to red represents low to high B-factors, respectively. Cα worm thickness also corresponds to B-factor magnitude, with low and high B-factors corresponding to thinner and thicker worms, respectively. (C) Figure shows representative electron density for the two Hairless molecules within the asymmetric unit. Su(H) is represented as a gray surface and the Hairless molecules (MBP/H-A and MBP/H-B) are shown in a stick representation with carbon, oxygen, and nitrogen atoms colored yellow, red, and blue, respectively. Electron density (slate blue mesh) corresponds to a simulated annealing composite omit map contoured at 1σ.
(PNG)

S2 Fig Sequence alignment of CSL orthologs. Figure shows sequence alignment of CSL orthologs from *D*. *melanogaster*, mouse, and human. Numbering corresponds to the fly ortholog Su(H). Sequences that correspond to the NTD, BTD, and CTD of CSL are colored cyan, green, and orange, respectively. The β-strand that makes hydrogen-bonding interactions with all three domains is colored magenta. Secondary structure elements for the CTD are shown above the sequence and are derived from the *apo* structure of mouse CSL (RBP-J) (3BRG). Circles (open and filled) denote residues in the CTD of Su(H) that contact Hairless in the complex structure, with the filled circles representing primarily side chain contacts and the open circles representing only main chain contacts. Overall, within the structural core of CSL, the primary sequence of Su(H) is 82% identical (90% similarity) to RBP-J; within the CTD, there is 75% identicalness (88% similarity) between fly and mouse CSL proteins. Of the 33 residues that are different between fly and mouse CSL, 27 of these residues (82%) have side chains that are surface exposed. Only 6 of the 33 residues that are different are buried or partially buried in the apo structure of CSL (3BRG), and in these cases the amino acid changes are very conservative, e.g., leucine to methionine, threonine to valine, isoleucine to valine, and serine to threonine.
(PNG)

S3 Fig Computational analysis of the Su(H)-Hairless structure. (A) Figure shows two independent MD simulations of the Su(H) structure after Hairless is removed from the complex, using the starting structure as the point of reference. The *x*- and *y*-axes correspond to the time (picoseconds) and the cα RMSD (root mean square deviation), respectively. (B) Figure shows the cα RMSF (root mean square fluctuation), as a function of residue number, for the two Su(H) simulations (red and black), and for comparative purposes, to a simulation for an *apo* structure of CSL (green, PDB ID: 3BRG) [9]. The black circle denotes large fluctuations in the CTD of Su(H), compared to *apo* CSL, which are indicative of large rearrangements in the CTD of Su(H) when Hairless is removed from the complex. (C) Figure shows cavity calculations (denoted at gray spheres with accompanying volume in Å^3^) of the CTD of Su(H) (colored orange, ribbon diagram) for the most representative structures during the time course of the MD simulations: “Start” refers to the first frame of the MD simulations; “Cluster 1, 2, and 3” describes the three most representative structures of the MD simulations during the early, middle, and late frames, respectively; and “Final” represents the final frame of the MD simulations. (D) Figure shows color-coded, time-based projection of the first eigenvector/eigenvalue for the PCA (principal component analysis) of the Su(H) structure from the MD simulations. The blue structure represents the first frame of the MD simulations and the red structure indicates the final frame. (E) Figure shows time-based F516 dihedral angle calculation during the time course of the two Su(H) simulations (red and green), as compared to the simulation of *apo* CSL (blue). As shown in the inset, F516 occupies two distinct conformations: (1) when bound to Hairless (yellow structure, corresponding to ~-50° dihedral angle) or (2) unbound (green structure, corresponding to ~50° dihedral angle). Very early in the Su(H) simulations (red and green), when Hairless is removed from the complex F516 immediately assumes a dihedral angle similar to the *apo* structure of CSL (blue), consistent with F516 assuming a high energy conformer in the presence of Hairless, which is rarely sampled.
(PNG)

S4 Fig Stability/Folding analysis of the Su(H) mutants. (A) Figure shows far UV circular dichroism data for purified recombinant Su(H) (98–523), wild-type and mutants. The NMRSD (normalized root-mean-square deviation) parameter values for analysis of the CD data were 0.161, 0.240, 0.305, 0.283, 0.255, and 0.128 for wild-type, L445A, L514A, F516A, L445A/L514A, L445A/F516A Su(H) proteins, respectively. The underlying data can be found in S1 Data. (B) Figure shows relative amounts of secondary-structure determined from CD data using Contin-LL (Provencher and Glockner Method) [31] with the SMP180 reference set for wild-type and mutant Su(H) constructs. (C) Figure shows thermal shift assays for wild-type and mutant Su(H) constructs. The underlying data can be found in S1 Data. (D) Figure shows representative EMSA for wild-type Su(H), and double mutants L445A/L514A and L445A/F516A, binding to an oligonucleotide duplex DNA containing a single CSL binding site. All lanes contain 1 uM DNA, and either 0.1, 0.5, or 1.0 uM of wild-type Su(H) or mutants, as indicated on the gel. Lane 1 DNA control, lanes 2–4 wild-type Su(H), lanes 5–7 Su(H) mutant L445A/L514A, and lanes 8–10 Su(H) mutant L445A/F516A.
(PNG)

S5 Fig Effects on eye morphology by the overexpression of Su(H) variants. Wild-type or mutant Su(H) protein variants as indicated were overexpressed singly or in combination with full-length Hairless in the eye imaginal disc using the *gmr-Gal4* driver line. UAS-GFP served as control. Crosses were kept at 25°C and resultant adult eyes are shown. Ectopic expression of wild-type Su(H), as well as the mutant isoforms Su(H)^LL/AA^, Su(H)^LLF/AAA^, and Su(H)^LLL/AAA^, affects eye morphology: eyes appear more bulgy and have slightly irregular ommatidia. Animals that only overexpress Hairless have smaller eyes with a rough appearance [63,64]. Co-overexpression of both Su(H) and Hairless leads to a strong reduction or a complete loss of the eye, and the flies die as pharate adults in their pupal case. Lethality is also observed when Hairless is combined with Su(H)^LL/AA^; however, the flies are able to eclose if incubated at 18°C. In contrast, the combined overexpression of the triple mutants Su(H)^LLF/AAA^ or Su(H)^LLL/AAA^ with Hairless is viable, and the eye phenotype is normalized. Notably, the combination of Hairless with Su(H)^LLL/AAA^ results in an almost normal fly, indicative of a complete lack of Hairless binding by this mutant.
(PDF)

S1 Table ITC data for the binding of Su(H) mutants to Hairless. All experiments were performed at 25°C. For native Su(H)-Hairless binding studies, table values are from Table 2 and are the mean of at least three independent experiments, and errors represent the standard deviation of multiple experiments. For all other table entries, the values represent ΔG_obs_, ΔH_obs_, and -TΔS_obs_ and the errors represent the standard deviation of the nonlinear least squares fit of the data to the titration curves. N/A = not applicable; for Su(H) mutants F460A and I500A we were unable to purify the recombinant protein for ITC binding studies.
(DOC)

## Abbreviations

ANK: ankyrin repeats
BTD: β-trefoil domain
CBF1: C-promoter binding factor 1
CBP: CREB binding protein
CD: circular dichroism
CSL: CBF1/Su(H)/Lag-1
CtBP: C-terminal binding protein
CTD: C-terminal domain
DSL: Delta/Serrate/Lag-2
H: Hairless
Ig: immunoglobulin
Lag-1: *lin-12* and *glp-1*
MAM: Mastermind
MBP: maltose binding protein
MD: molecular dynamics
MINT: MSX-2 interacting nuclear target
NICD: Notch intracellular domain
NTD: N-terminal domain
RAM: RBP-J associated molecule
SER: surface entropy reduction
SHARP: SMRT/HDAC1 associated repressor protein
Su(H): Suppressor of Hairless
Y2H: yeast two-hybrid
