# Supplementary figures and images for: Structure and Function of the Su(H)-Hairless Repressor Complex, the Major Antagonist of Notch Signaling in Drosophila melanogaster
Source: PLoS Biol. 2016 Jul 12;14(7):e1002509. doi: 10.1371/journal.pbio.1002509 (PMC4942083; doi:10.1371/journal.pbio.1002509)

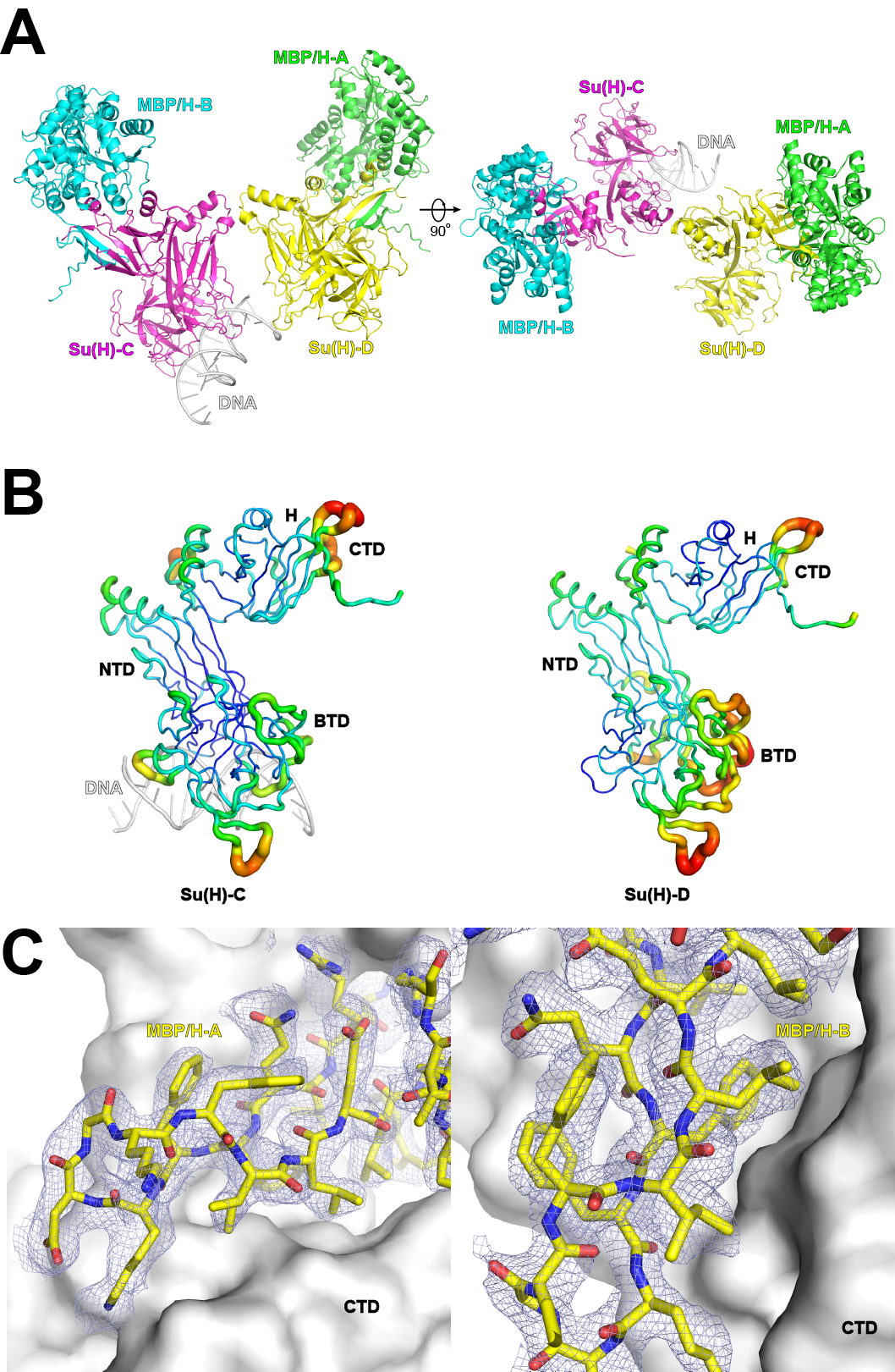

Supplement: S1 Fig — (A) Figure shows orthogonal views of the molecules contained within the asymmetric unit of the crystal. The asymmetric unit contains two Su(H) molecules (colored yellow and magenta), two MBP-Hairless fusion molecules (colored cyan and green), and one DNA duplex (colored gray). MBP/H-A, MBP/H-B, Su(H)-C, and, Su(H)-D refer to chains A, B, C, and D, respectively, contained within the PDB file. (B) Figure shows a temperature factor (B-factor) comparison of the two Su(H) molecules contained within the asymmetric unit of the crystals. The color gradient from blue to red represents low to high B-factors, respectively. Cα worm thickness also corresponds to B-factor magnitude, with low and high B-factors corresponding to thinner and thicker worms, respectively. (C) Figure shows representative electron density for the two Hairless molecules within the asymmetric unit. Su(H) is represented as a gray surface and the Hairless molecules (MBP/H-A and MBP/H-B) are shown in a stick representation with carbon, oxygen, and nitrogen atoms colored yellow, red, and blue, respectively. Electron density (slate blue mesh) corresponds to a simulated annealing composite omit map contoured at 1σ. (PNG) [file pbio.1002509.s002.png]

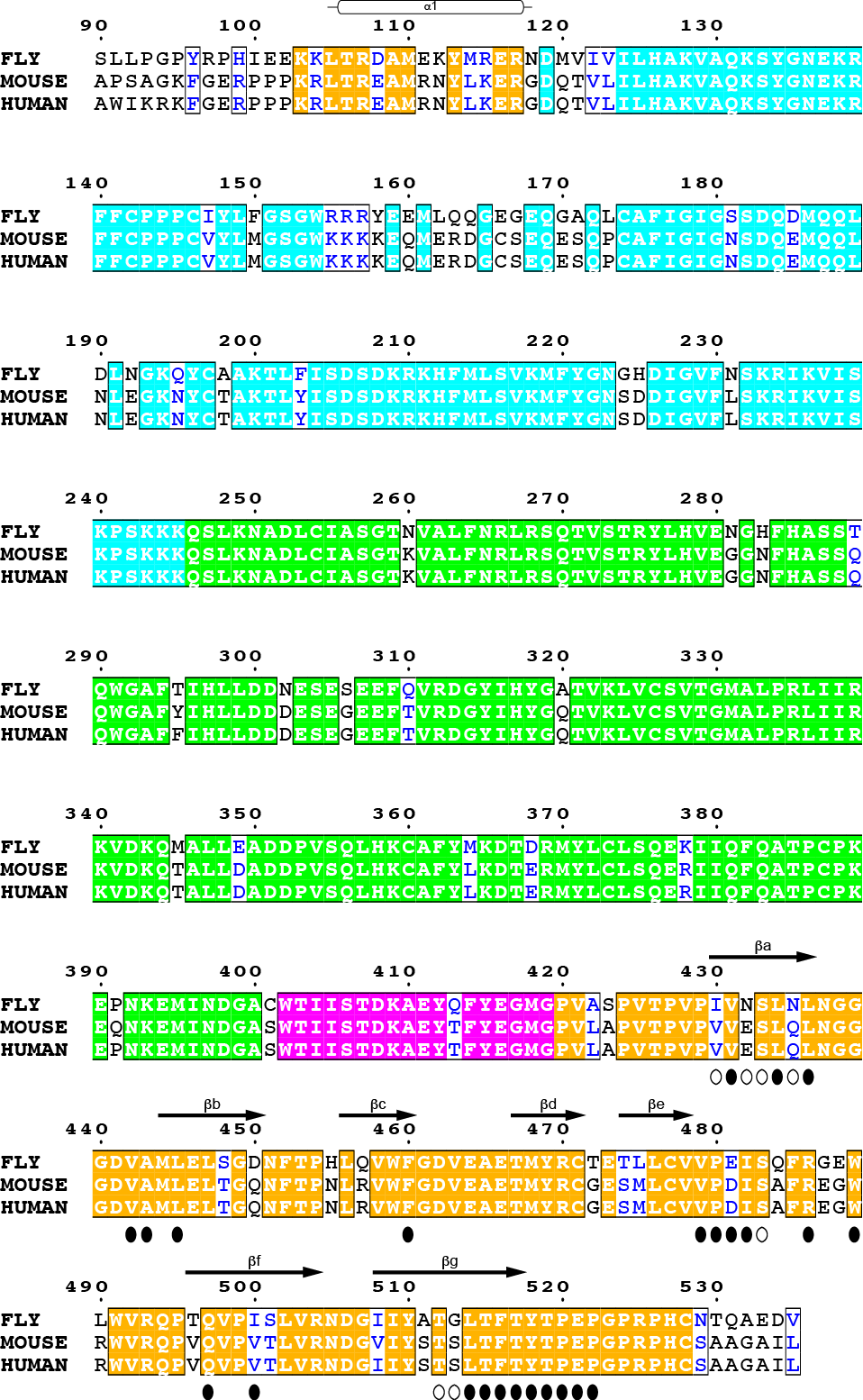

Supplement: S2 Fig — Figure shows sequence alignment of CSL orthologs from D. melanogaster, mouse, and human. Numbering corresponds to the fly ortholog Su(H). Sequences that correspond to the NTD, BTD, and CTD of CSL are colored cyan, green, and orange, respectively. The β-strand that makes hydrogen-bonding interactions with all three domains is colored magenta. Secondary structure elements for the CTD are shown above the sequence and are derived from the apo structure of mouse CSL (RBP-J) (3BRG). Circles (open and filled) denote residues in the CTD of Su(H) that contact Hairless in the complex structure, with the filled circles representing primarily side chain contacts and the open circles representing only main chain contacts. Overall, within the structural core of CSL, the primary sequence of Su(H) is 82% identical (90% similarity) to RBP-J; within the CTD, there is 75% identicalness (88% similarity) between fly and mouse CSL proteins. Of the 33 residues that are different between fly and mouse CSL, 27 of these residues (82%) have side chains that are surface exposed. Only 6 of the 33 residues that are different are buried or partially buried in the apo structure of CSL (3BRG), and in these cases the amino acid changes are very conservative, e.g., leucine to methionine, threonine to valine, isoleucine to valine, and serine to threonine. (PNG) [file pbio.1002509.s003.png]

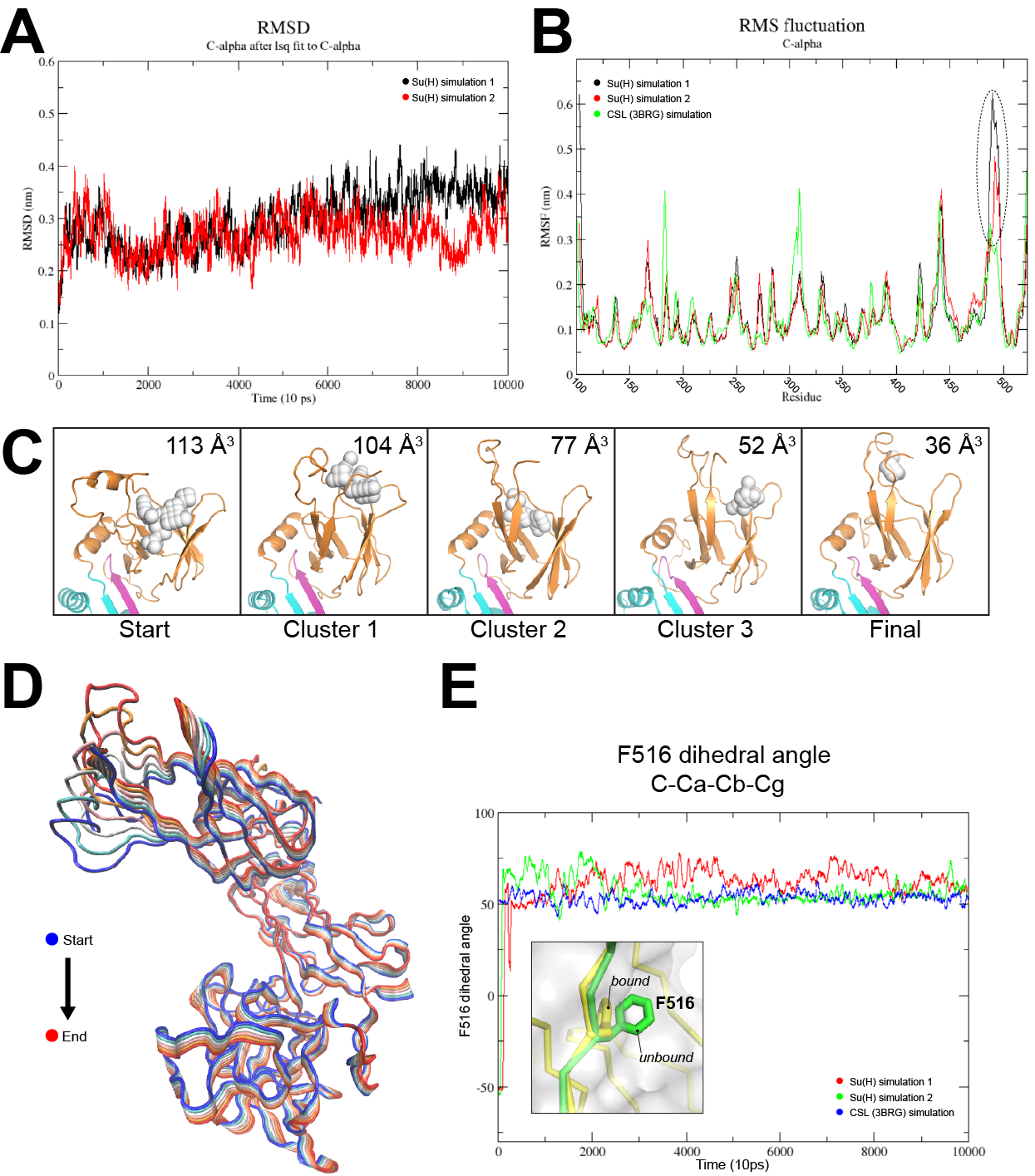

Supplement: S3 Fig — (A) Figure shows two independent MD simulations of the Su(H) structure after Hairless is removed from the complex, using the starting structure as the point of reference. The x- and y-axes correspond to the time (picoseconds) and the cα RMSD (root mean square deviation), respectively. (B) Figure shows the cα RMSF (root mean square fluctuation), as a function of residue number, for the two Su(H) simulations (red and black), and for comparative purposes, to a simulation for an apo structure of CSL (green, PDB ID: 3BRG) [9]. The black circle denotes large fluctuations in the CTD of Su(H), compared to apo CSL, which are indicative of large rearrangements in the CTD of Su(H) when Hairless is removed from the complex. (C) Figure shows cavity calculations (denoted at gray spheres with accompanying volume in Å3) of the CTD of Su(H) (colored orange, ribbon diagram) for the most representative structures during the time course of the MD simulations: “Start” refers to the first frame of the MD simulations; “Cluster 1, 2, and 3” describes the three most representative structures of the MD simulations during the early, middle, and late frames, respectively; and “Final” represents the final frame of the MD simulations. (D) Figure shows color-coded, time-based projection of the first eigenvector/eigenvalue for the PCA (principal component analysis) of the Su(H) structure from the MD simulations. The blue structure represents the first frame of the MD simulations and the red structure indicates the final frame. (E) Figure shows time-based F516 dihedral angle calculation during the time course of the two Su(H) simulations (red and green), as compared to the simulation of apo CSL (blue). As shown in the inset, F516 occupies two distinct conformations: (1) when bound to Hairless (yellow structure, corresponding to ~-50° dihedral angle) or (2) unbound (green structure, corresponding to ~50° dihedral angle). Very early in the Su(H) simulations (red and green), when Hairless is removed f [file pbio.1002509.s004.png]

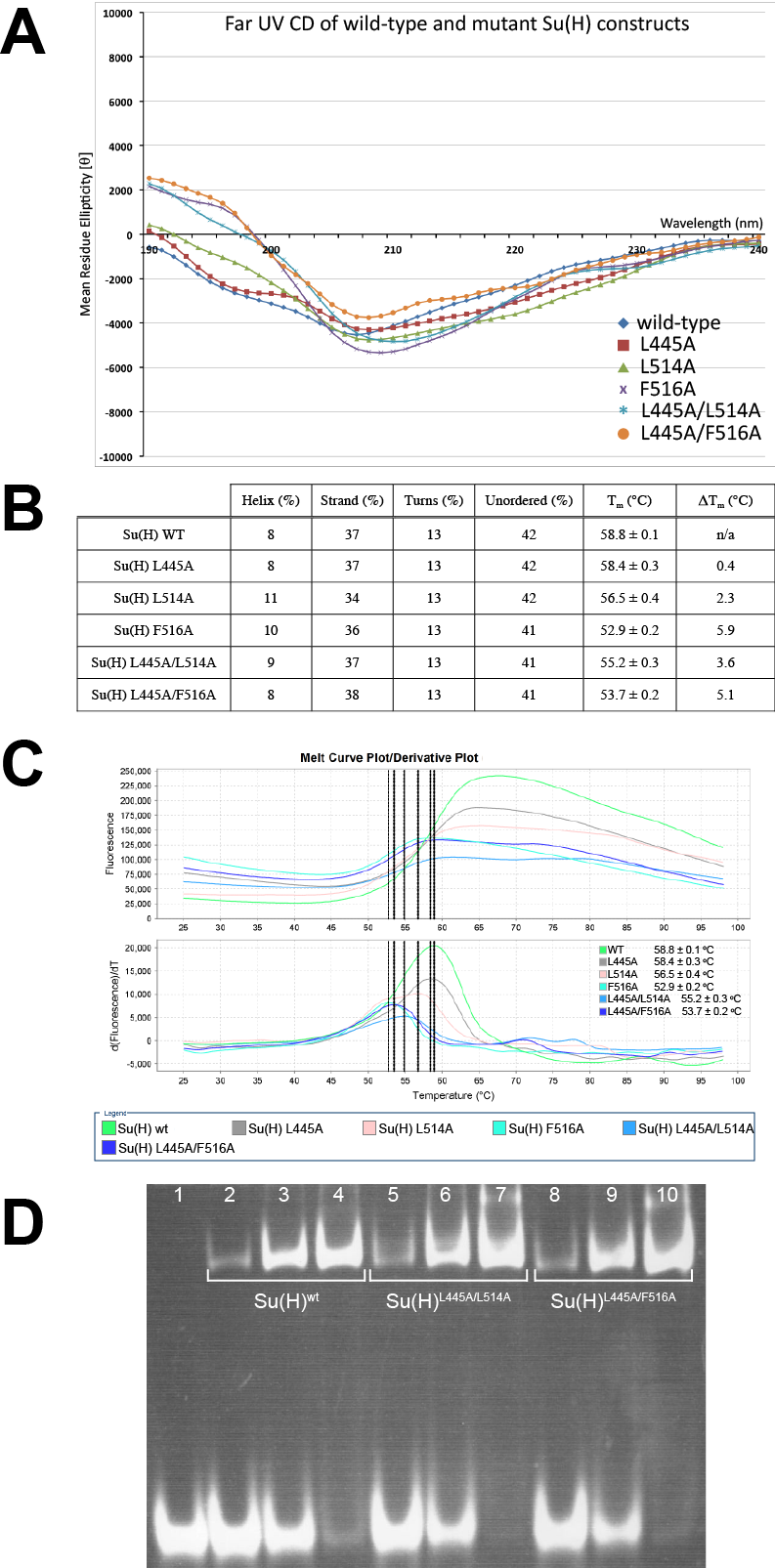

Supplement: S4 Fig — (A) Figure shows far UV circular dichroism data for purified recombinant Su(H) (98–523), wild-type and mutants. The NMRSD (normalized root-mean-square deviation) parameter values for analysis of the CD data were 0.161, 0.240, 0.305, 0.283, 0.255, and 0.128 for wild-type, L445A, L514A, F516A, L445A/L514A, L445A/F516A Su(H) proteins, respectively. The underlying data can be found in S1 Data. (B) Figure shows relative amounts of secondary-structure determined from CD data using Contin-LL (Provencher and Glockner Method) [31] with the SMP180 reference set for wild-type and mutant Su(H) constructs. (C) Figure shows thermal shift assays for wild-type and mutant Su(H) constructs. The underlying data can be found in S1 Data. (D) Figure shows representative EMSA for wild-type Su(H), and double mutants L445A/L514A and L445A/F516A, binding to an oligonucleotide duplex DNA containing a single CSL binding site. All lanes contain 1 uM DNA, and either 0.1, 0.5, or 1.0 uM of wild-type Su(H) or mutants, as indicated on the gel. Lane 1 DNA control, lanes 2–4 wild-type Su(H), lanes 5–7 Su(H) mutant L445A/L514A, and lanes 8–10 Su(H) mutant L445A/F516A. (PNG) [file pbio.1002509.s005.png]
