# Supplementary material for: Structure and Function of the Su(H)-Hairless Repressor Complex, the Major Antagonist of Notch Signaling in Drosophila melanogaster
Source: PLoS Biol. 2016 Jul 12;14(7):e1002509. doi: 10.1371/journal.pbio.1002509 (PMC4942083; doi:10.1371/journal.pbio.1002509)
Supplement: S5 Fig — Wild-type or mutant Su(H) protein variants as indicated were overexpressed singly or in combination with full-length Hairless in the eye imaginal disc using the gmr-Gal4 driver line. UAS-GFP served as control. Crosses were kept at 25°C and resultant adult eyes are shown. Ectopic expression of wild-type Su(H), as well as the mutant isoforms Su(H)LL/AA, Su(H)LLF/AAA, and Su(H)LLL/AAA, affects eye morphology: eyes appear more bulgy and have slightly irregular ommatidia. Animals that only overexpress Hairless have smaller eyes with a rough appearance [63,64]. Co-overexpression of both Su(H) and Hairless leads to a strong reduction or a complete loss of the eye, and the flies die as pharate adults in their pupal case. Lethality is also observed when Hairless is combined with Su(H)LL/AA; however, the flies are able to eclose if incubated at 18°C. In contrast, the combined overexpression of the triple mutants Su(H)LLF/AAA or Su(H)LLL/AAA with Hairless is viable, and the eye phenotype is normalized. Notably, the combination of Hairless with Su(H)LLL/AAA results in an almost normal fly, indicative of a complete lack of Hairless binding by this mutant. (PDF) [file pbio.1002509.s006.pdf]

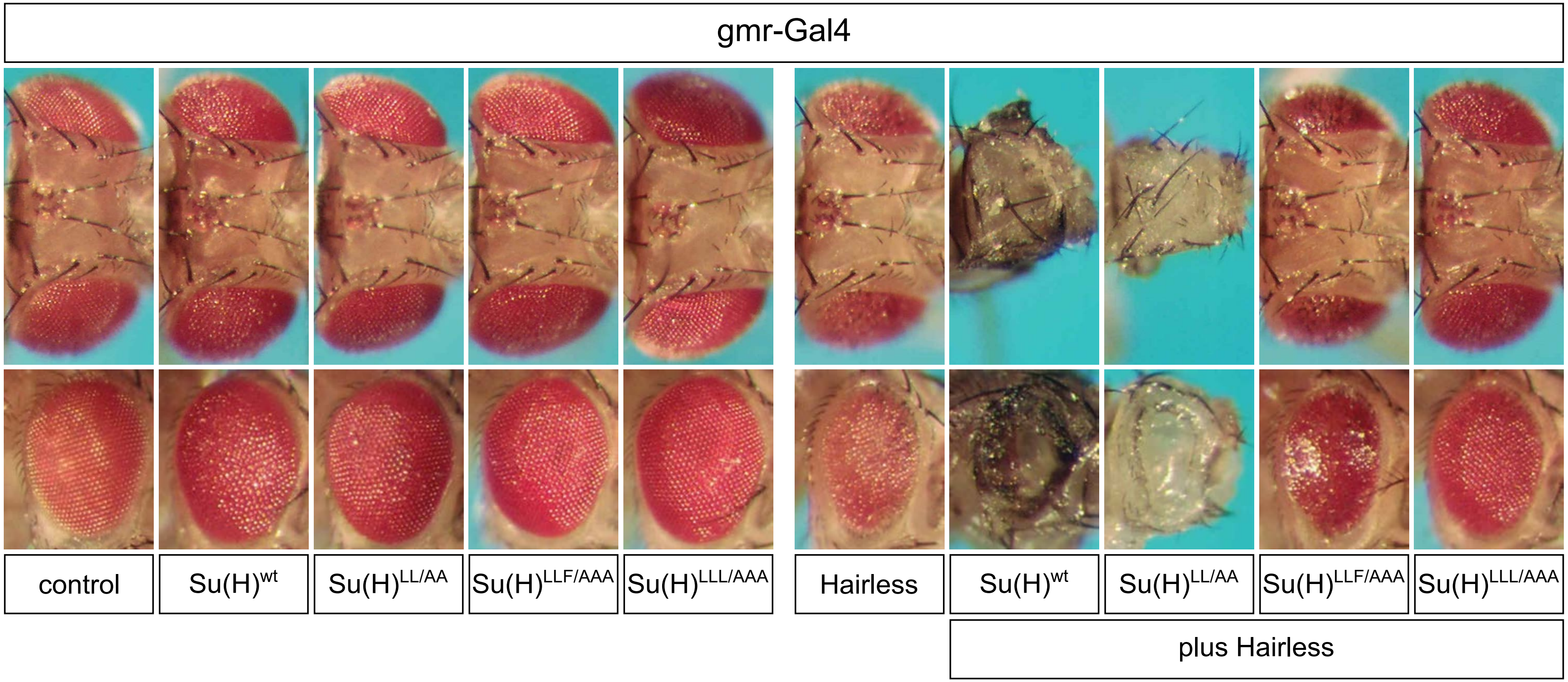
gmr-Gal4
control
Su(H)wt
Su(H)LL/AA
Su(H)LLF/AAA
Su(H)LLL/AAA
Hairless
Su(H)wt
Su(H)LL/AA
Su(H)LLF/AAA
Su(H)LLL/AAA
plus Hairless
