# Supplementary material for: Structure and Function of the Su(H)-Hairless Repressor Complex, the Major Antagonist of Notch Signaling in Drosophila melanogaster
Source: PLoS Biol. 2016 Jul 12;14(7):e1002509. doi: 10.1371/journal.pbio.1002509 (PMC4942083; doi:10.1371/journal.pbio.1002509)
Supplement: S1 Table — All experiments were performed at 25°C. For native Su(H)-Hairless binding studies, table values are from Table 2 and are the mean of at least three independent experiments, and errors represent the standard deviation of multiple experiments. For all other table entries, the values represent ΔGobs, ΔHobs, and -TΔSobs and the errors represent the standard deviation of the nonlinear least squares fit of the data to the titration curves. N/A = not applicable; for Su(H) mutants F460A and I500A we were unable to purify the recombinant protein for ITC binding studies. (DOC) [file pbio.1002509.s007.doc]

**S1 Table: ITC data for the binding of Su(H) mutants to Hairless.**

| *Cell* | *Syringe* | *K (M^-1^)* | *K_d_ (μM)* | *ΔG° (kcal/mol)* | *ΔH° (kcal/mol)* | *-TΔS° (kcal/mol)* |
| --- | --- | --- | --- | --- | --- | --- |
| Su(H) | Hairless | 5.7 ± 1.7 x 10^8^ | 0.002 | -11.9 ± 0.2 | -11.9 ± 0.4 | -0.05 ± 0.6 |
| Su(H)^L434A^ | Hairless | 6.4 ± 0.9 x 10^8^ | 0.002 | -12.0 | -11.9 ± 0.3 | -0.1 |
| Su(H)^L436A^ | Hairless | 5.0 ± 0.5 x 10^8^ | 0.002 | -11.9 | -13.0 ± 0.3 | 1.1 |
| Su(H)^V479A^ | Hairless | 4.5 ± 0.2 x 10^8^ | 0.002 | -11.8 | -13.2 ± 0.2 | 1.4 |
| Su(H)^I482A^ | Hairless | 1.8 ± 1.5 x 10^9^ | 0.001 | -12.6 | -16.9 ± 0.2 | 4.3 |
| Su(H)^F516A^ | Hairless | 7.5 ± 0.4 x 10^8^ | 0.001 | -12.1 | -14.2 ± 0.2 | 2.1 |
| Su(H)^Y518A^ | Hairless | 1.7 ± 1.1 x 10^9^ | 0.001 | -12.6 | -16.6 ± 0.2 | 4.0 |
| Su(H)^F460A^ | Hairless | N/A | N/A | N/A | N/A | N/A |
| Su(H)^I500A^ | Hairless | N/A | N/A | N/A | N/A | N/A |
